# Herb–drug interactions between the medicinal mushrooms Lingzhi and Yunzhi and cytotoxic anticancer drugs: a systematic review

**DOI:** 10.1186/s13020-020-00356-4

**Published:** 2020-07-25

**Authors:** Chun Sing Lam, Lok Pui Cheng, Li Min Zhou, Yin Ting Cheung, Zhong Zuo

**Affiliations:** 1 School of Pharmacy, Faculty of Medicine, The Chinese University of Hong Kong, Shatin, N.T., Hong Kong, People’s Republic of China; 2 School of Chinese Medicine, Hong Kong Baptist University, Kowloon City, Hong Kong, People’s Republic of China

**Keywords:** Herb–drug interaction, Lingzhi, Yunzhi, Cytotoxic drugs, Anticancer drugs, Medicinal mushrooms

## Abstract

**Background:**

Lingzhi and Yunzhi are medicinal mushrooms commonly used with cytotoxic chemotherapy in cancer patients in Asian countries. The current systematic review aims to identify potential pharmacokinetic or pharmacodynamic interactions from the existing literature to ensure their effective and safe combination usage in cancer patients.

**Methods:**

A systematic search was conducted on nine major Chinese and English databases, including China Journal Net, Allied and Complementary Medicine Database, and Ovid MEDLINE^®^, etc., to identify clinical, animal, and in-vitro studies that evaluate the effect of combined use of Lingzhi or Yunzhi with cytotoxic drugs. The Jadad scale was used to assess the quality of clinical studies.

**Results:**

This search identified 213 studies, including 77 clinical studies that reported on the combined use of cytotoxic drugs with Yunzhi (n = 56) or Lingzhi (n = 21). Majority of these clinical studies demonstrated modest methodological quality. In clinical practice, the most commonly used cytotoxic drugs with Lingzhi were cisplatin, 5-fluorouracil (5-FU) and paclitaxel, whereas Tegafur/uracil (UFT)/Tegafur, 5-FU, and mitomycin were the ones used more often with Yunzhi. Only two clinical pharmacokinetic studies were available showing no significant interactions between Polysaccharide K (PSK) and Tegafur. From the pharmacodynamic interactions perspective, combination uses of Yunzhi/Lingzhi with cytotoxic drugs in clinical practice could lead to improvement in survival (n = 31) and quality of life (n = 17), reduction in tumor lesions (n = 22), immune modulation (n = 38), and alleviation of chemotherapy-related side effects (n = 14) with no reported adverse effects.

**Conclusion:**

Our findings suggest that the clinical combination use of Lingzhi or Yunzhi with cytotoxic drugs could enhance the efficacy and ameliorate the adverse effects of cytotoxic drugs, leading to improved quality of life in cancer patients. More high quality clinical studies including pharmacokinetic herb-drug interactions studies are warranted to verify these observations and mechanisms involved. Based on the high quality clinical data, pharmacoepidemiology methods and bioinformatics or data mining could be adopt for further identification of clinical meaningful herb-drug interactions in cancer therapies.

## Background

Although chemotherapy and radiotherapy remain the mainstay of cancer treatment in developed countries, an increasing number of cancer patients are seeking benefits from complementary and alternative medicine. Surveys in the United States, Canada and Europe revealed that an average rate of 35% of cancer patients have utilized Chinese herbal medicine during their treatment [1]. Such prevalence of Chinese herbal medicine use in cancer patients from Asian countries is expected to be even higher [2, 3].

It is well-known that the concurrent use of Chinese and Western medicines can cause herb-drug interactions that lead to both beneficial and harmful health outcomes. To highlight, herb–drug interactions are not uncommon in cancer treatment and may affect the clinical efficacy or safety of the treatment. One study demonstrated that over half of the patients undergoing chemotherapy took herbal products, of whom 27% were found to be at risk of clinically significant interactions between chemotherapy drug and herbs. In another study, authors detected 120 possible herb–drug interactions in 149 patients who reported concurrent use of Chinese herbs with conventional anti-cancer drugs [4, 5]. As most chemotherapy drugs have a narrow therapeutic index, there is an urging need for clinicians and scientists to address the potential herb-drug interactions in oncology practice.

Among Chinese herbs, medicinal mushrooms have been used for a long time during the cancer treatment. Lingzhi (Reishi or Mannentake in Japanese) and Yunzhi (commonly known as Turkey tail) are common medicinal mushrooms that are readily available in Asian countries. They are believed to possess medicinal properties to treat cancers or relieve cancer-related symptoms [6]. The two mushrooms both belong to the Polyporaceae family and have similar characteristics based on Traditional Chinese Medicine theory including flavour and nature [7]. Despite their popular use in cancer patients, there are limited reports on the clinical outcomes from their herb-drug interactions during anti-cancer treatment.

Although systematic reviews and meta-analysis of Lingzhi and Yunzhi as an adjunct for cancer treatment have been performed [8–10], these reviews mostly focused on clinical outcomes with no mechanistic explanations for the potential beneficial or harmful interactions. By gathering both clinical and preclinical studies of this subject matter, the current systematic review aimed to evaluate the effects of the co-administration of cytotoxic drugs with the medicinal mushrooms, Lingzhi and Yunzhi. Specifically, we will identify potential pharmacokinetic and pharmacodynamic interactions between these medicinal mushrooms and chemotherapy drugs and discuss the implications of these interactions on the efficacy and safety of cancer treatment.

## Methodology

### Database search

A comprehensive search was conducted on the following databases: China Journal Net (1915 to June 2020), Wanfang Database (1990 to June 2020), and Chinese Biomedical Literature Database (1878 to June 2020). English databases included Allied and Complementary Medicine (1985 to June 2020), Embase (1910 to June 2020), Ovid MEDLINE^®^ (1946 to June 2020), Ovid Nursing Database (1946 to June 2020), Ovid Emcare (1995 to June 2020), and Natural Medicines Comprehensive Database.

The combination of search terms included keywords for cytotoxic anticancer drugs and medicinal mushrooms as shown in Additional file 1: Table S1. The keywords used for cytotoxic drugs were based on the Hong Kong Hospital Authority Drug Formulary with no targeted therapy drugs included in the current review. In addition, chemoprotectants such as leucovorin and mesna were included in the current search. Besides the specific names of the cytotoxic drugs, general terms such as “cytotoxic drug” and “antineoplastic drug” were also included in the search to increase the coverage.

For the two medicinal mushrooms, Chinese name, English name, Pinyin and Latin name of them together with the names of their active ingredients were incorporated in the search. For Lingzhi, keywords for search included Reishi, Mannentake, Lingzhi, *Ganoderma lucidum*, *Ganoderma sinense*, Ganoderic acid, Polysaccharide, 靈芝, 靈芝酸, and 多糖. For Yunzhi, Turkey Tail, Yunzhi, *Coriolus versicolor*, *Trametes versicolor*, *Polyporus versicolor*, Krestin (PSK), Polysaccharide, Polysaccharide peptide (PSP), 雲芝 and 多糖肽. General terms including “medicinal mushroom” was also utilized in the search for more comprehensive coverage.

### Inclusion criteria

This review included clinical, animal and in-vitro studies that reported the concurrent use of any cytotoxic drug with the two medicinal mushroom(s). The name of drug used, dosage form and administration route should be specified. The mushroom(s) could exist in any formulation containing a raw or processed form of the mushroom(s) that included the extract of its (their) active phytochemical components. They could be used alone or with other herbs or ingredients in a composite formula. Clinical studies had to involve two groups of patients, a control group that received only the cytotoxic drug or the medicinal mushroom(s) (or the mushroom-containing herbal formula) and a co-administration group that received the cytotoxic drug together with the medicinal mushroom(s). If the control group also received medicinal mushrooms, there should be significant dosage differences of the mushrooms in the treatment group. The languages of the included articles were restricted to English, Chinese and Japanese.

### Assessing the quality of clinical studies

The Jadad scale was used to assess the quality of the clinical studies. This is a 5-point scale evaluating randomization, blinding, and withdrawals or dropouts of the clinical trials [11].

## Results and discussion

### Results of the database search

**Fig. 1:**
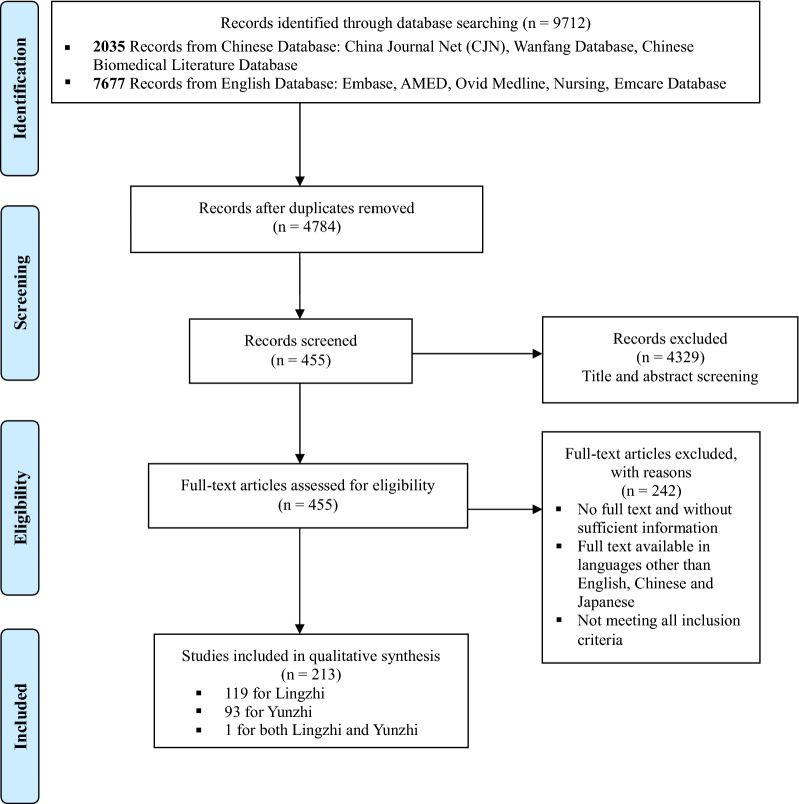
PRISMA flow chart of current database search and literature selection

Figure 1 summarizes the process of database searching and literature selection. The initial search identified 9712 studies. After screening their titles and abstracts, about 455 studies were included and subjected to further full-text inspection and verifications based on the inclusion criteria. In total, 213 studies were included in this review with 119 of them focused on Lingzhi, 93 of them on Yunzhi, and 1 on the combination of Lingzhi and Yunzhi. Other characteristics of the included studies including languages used, type of studies, type of the mushroom preparations, and country of the origin, type of cancer together with Jadad score of the included clinical studies were summarized in Additional file 1: Table S2 and distributions of the included cytotoxic drugs were shown Additional file 1: Figure S1. The detailed information on the 77 clinical studies and 137 preclinical studies were listed in Additional file 1: Tables S3, S4, respectively.

### Interaction outcome

#### Pharmacokinetic interactions

In general, evidence on the pharmacokinetic interactions between Lingzhi or Yunzhi and cytotoxic drugs was scarce, except for the clinical pharmacokinetics interaction investigations between PSK and Tegafur as illustrated below.

##### Clinical studies on pharmacokinetic interaction

Of all the 213 studies included in this review, only two studies evaluated the clinical pharmacokinetic interactions between cytotoxic drugs and medicinal mushrooms. Both studies evaluated the interactions between PSK (Yunzhi) and Tegafur (tetrahydrofuryl-5-fluorouracil), the prodrug of cytotoxic drug 5-FU. The conversion of Tegafur to 5-FU is mediated primarily by the CYP2A6 enzyme. Only small or non-significant changes in the blood level of 5-FU and Tegafur was observed in most patients with up to 8–14 months PSK co-administration [12, 13].

##### Pre-clinical studies on pharmacokinetic interaction

Only two in-vitro studies included in the review evaluated the pharmacokinetic interactions between Lingzhi and cytotoxic drugs. Ganoderic polysaccharides were shown to increase the distribution of doxorubicin in drug-resistant cancer cell lines, while Ganoderic acid B was shown to reverse the resistance of hepatocellular carcinoma toward doxorubicin, paclitaxel, and vincristine but not cisplatin [14, 15]. Since doxorubicin, paclitaxel, and vincristine are substrates of P-glycoprotein, Lingzhi or its components were expected to modulate the P-gp mediated efflux of these cytotoxic drugs to increase their cell accumulation and reverse their resistance in the tumor cells [16].

For Yunzhi, consistent with clinical findings, an animal study showed that regardless of the administration route, PSK had no effect on the tissue level of 5-FU or its prodrug Tegafur in tumor-bearing mice [17]. In addition, another animal study showed that PSP could decrease cyclophosphamide clearance and increase the plasma half-life without affecting its distribution or protein binding. It was also found that a dose-dependent relationship existed between the systemic exposure of cyclophosphamide and acute rather than sub-chronic treatment with PSP [18].

#### Pharmacodynamic interaction

Tables 1 and 2 summarized the clinical and preclinical pharmacodynamic interactions between Lingzhi/Yunzhi and cytotoxic drugs, respectively. Outcomes on survival and quality of life, tumor inhibition and disease control rate, immune system and alleviation of the other adverse effects of cytotoxic drugs were further illustrated as follows.

**Table 1:** Pharmacodynamic interactions between medicinal mushrooms and cytotoxic drugs in clinical studies

Drugs	Interactions with Lingzhi	Interactions with Yunzhi
5-FU	Increase in clinical efficacy [19, 52, 59, 76], survival time [19] and quality of life [52, 59] Better symptomatic relief [65, 139], less adverse effects (gastrointestinal, stomatitis) [19, 59] Less decrease in WBC [19, 52, 59], including T cells (CD4+, CD4+/CD8+ ratio) [76, 134], NK cells [52, 134], platelets [59], hemoglobin [52] Decrease level of miR-21, endoglin, TGF-*β*1 and VEGF in tumor tissue [76]	Increase in survival [22–30], reduce recurrence [25] and quality of life [60] Less decrease in WBC, hemoglobin, platelets [136–138] Increase in CD3+, CD4+, CD4+/CD8+, decrease in CD8+ T cells [60, 136, 138–140], increase in NK cell activity [23, 60, 136, 139, 140], LBT [138], IL-2 [136] Reduce adverse effects (fatigue, nausea/vomiting) [136]
Capecitabine	Increase in disease control rate and quality of life [53] Less decrease in WBC [53, 132], nausea and vomiting [53]	Improve disease progression, survival and quality of life [48] Reduce adverse effects (BMS, mucositis, hand-foot syndrome, diarrhea) [48]
Carboplatin	No studies available	Increase in efficacy, quality of life [49, 62], survival, and reduce metastasis [49] Better symptomatic relief [49] and relieve WBC decrease [49, 62] Increase in CD3+, CD4+ and decrease in CD8+ T cells [49, 62] Reduction in tumor cell markers and invasive cell factors, such as VEGF, MMP-9, CEA, sMICA [62]
Cisplatin	Increase in clinical efficacy [20, 21, 52, 54–56, 76–78], survival [20] and quality of life [21, 52, 54–58] Reduce BMS [54, 56, 57, 77, 78] (increase in RBC [54], WBC [21, 52, 54, 56, 57], NK cells [52, 55], hemoglobin [52, 54], platelets [54, 56]), less decrease in CD3+, CD4+ T cells, CD4+/CD8+, less increase in CD8+ [20, 54, 55, 76, 77, 133] Better symptomatic relief [20, 21, 56] and less adverse effects (gastrointestinal, nausea/vomiting, anemia, renal damage, ALT increase) [56, 77, 78] Decrease level of miR-21, endoglin, TGF-β1 and VEGF in tumor tissue [76]	Increase in clinical efficacy [49, 79], increase in survival and lower metastasis [49] Increase in quality of life [49, 60] and better symptomatic relief [49, 141] Elevate CD3+, CD4+, CD4+/CD8+ [49, 60, 141, 142], NK cell activity [60, 141], immunoglobulin production [79], IL production [142] Reduce side effects (peripheral neuropathy, decrease in WBC) [143] Less weight loss [141]
Cyclophosphamide	No studies available	Increase in clinical efficacy and survival, lower metastasis [49] Increase quality of life [49, 60], better symptomatic relief [49], increase appetite [144] Less decrease in WBC, platelets [137, 138, 144], increase CD3+, CD4+, CD4+/CD8+, decrease CD8+ T cells, increase NK cells, LBT [49, 60, 138, 142], IL-2 production [142] Reduce sister chromatid exchange frequency [172]
Doxorubicin	Increase in efficacy and survival time [19] Better symptomatic relief [134], and less adverse effects (nausea, vomiting, diarrhea) [19] Less decrease in WBC [19], including T cells and NK cells [134]	Increase in survival [31, 49], efficacy [49, 80], quality of life [49, 60], reduce metastasis [49] Better symptomatic relief [49] and increase appetite [144] Relieve decrease in WBC (including T cells, NK cells) [60, 80, 144], platelet and hemoglobin [80, 140, 142, 144]. Increase in CD3+, CD4+, CD4+/CD8+ and decrease in CD8+ T cells [49, 60, 80, 140], reduce inhibition on IL-2 production [142]
Etoposide	No studies available	Increase in efficacy [49, 79], survival, quality of life and lower metastasis [49] Better symptomatic relief [49] Less decrease in WBC (including CD3+ T cells) [49, 68] and increase in immunoglobulin production [79]
Gemcitabine	Increase in efficacy and quality of life [21, 56] Better symptomatic relief [21, 56] and less adverse effects (anemia, gastrointestinal, nausea/vomiting, increase in ALT, renal damage) [56] Less decrease in WBC [21, 56], platelets [56]	No studies available
Leucovorin	Increase in efficacy [52, 59] and quality of life [52, 59] Less reduction in WBC [52, 59], NK cells [52], platelets [59], hemoglobin [52] Better symptomatic relief [59], reduce adverse events (gastrointestinal, nausea/vomiting, stomatitis) [59]	No benefits in survival [51]
Mercaptopurine	No studies available	Prolongation of complete remission and survival [50] Increase cell-mediated immunity [50]
Methotrexate	No studies available	Less decrease in WBC and platelet [137, 138] Less decrease in CD3+, CD4+, CD4+/CD8+ and LBT and greater decrease in CD8+ for PSK and selenium Yunzhi formulation, but not PSP [137, 138]
Mitomycin	Increase in efficacy and survival [19, 20] Better symptomatic relief [20, 134] and less adverse effects (nausea/vomiting, diarrhea) [19] Less decrease in WBC (T cell and NK cell) [19, 20, 134]	Increase in survival [24, 28, 31, 33–37], quality of life [60] and better symptomatic relief [141] Less decrease in WBC [136, 143], hemoglobin and platelets [136] Increase in CD3+, CD4+ T cells, CD4+/CD8+ ratio, NK cell activity and IL production, decrease in CD8+ T cells [60, 136, 139–141] Reduce adverse effects (peripheral neuropathy [143], nausea/vomiting, fatigue [136]) and less weight loss [141]
Oxaliplatin	Increase in clinical efficacy [53, 59], quality of life [53, 59] and better symptomatic relief [59] Relieve reduction in WBC [53, 59, 132] and platelets [59] Reduce side effects (gastrointestinal, nausea/vomiting, stomatitis) [53, 59]	Improve disease progression, survival and quality of life [48] Reduce adverse effects (BMS, mucositis, hand-foot syndrome, diarrhea) [48]
Paclitaxel	Increase in clinical efficacy [55, 56, 59, 77, 78], quality of life [55, 56, 58, 59] Reduce BMS (less anemia, reduction in WBC—T cells, NK cells, platelets) [55, 56, 59, 77, 78], increase in CD3+, CD4+, CD4+/CD8+, decrease in CD8+ [55, 77, 78, 133] Better symptomatic relief [56, 59] and reduce side effects (gastrointestinal, nausea, vomiting, stomatitis, renal damage, increase in ALT) [56, 59, 77, 78]	Increase in efficacy [62] and quality of life [62] Relieve decrease in CD3+, CD4+ T cells and increase in CD8+ T cells [62] Reduction in tumor cell markers and invasive cell factors, such as VEGF, MMP-9, CEA, sMICA [62]
UFT/Tegafur	No studies available	Increase in survival [23, 27, 29–32, 38–47] and reduce recurrence [38, 42, 44] Increase quality of life [61] and better symptomatic relief [32] Reduce adverse effects (appetite loss, nausea/vomiting, gastrointestinal) [61] Recover immunosuppression [47, 135], increase NK cells [23, 38] Decrease in suppressor T cells and increase cytotoxic T cells [38]. Reduction in CD57+ T cell [39]. Reduce T cell apoptosis and caspase 3 activity through downregulating Bax expression in T cells [170]
Vincristine	Increase in efficacy and survival [20], better symptomatic relief [20] increase in T cells count (CD4, CD4/CD8) [20]	Better symptomatic relief [141] and increase appetite [144] Less reduction in WBC (CD3+, CD4+ T cells, NK cells) [141, 144], platelets [144]
Vinorelbine	Increase quality of life [57] and reduce BMS (less decrease in WBC) [57]	No studies available

**Table 2:** Pharmacodynamic interactions between medicinal mushrooms and cytotoxic drugs in preclinical studies

Drugs	Type	Interactions with Lingzhi	Interactions with Yunzhi
5-FU	Animal	Increase in tumor inhibition effect [83, 84, 98] and survival time [63] Less decrease in WBC [83, 84, 145], platelets [83] and other bone marrow cells [84] Greater recovery rate from intestinal damage caused by 5-FU [173]	Increase in survival [67], cytotoxicity and inhibit metastasis [111] Reduce immunosuppression (reduce the decrease in phagocytic activity, antibody production [111], delayed hypersensitivity [111, 112])
	In-vitro	Increase in tumor inhibition and apoptotic rate (dose-related or time-dependent) [98–103], similar effect at lower dose of 5-FU [105] Induce cell cycle arrest at different phases [99, 101], increase caspase 3 and 8 expression and activity [100]. Increase release of cytochrome C [100, 102] and MMP [102] Increase level of DNA strand breaks and oxidative damage in cancer cells [98]	Enhance 5-FU cytotoxicity [118, 119] Decrease dihydropyrimidine dehydrogenase mRNA expression, and increase IFN-α mRNA expression [118]
Carboplatin	Animal	Less decrease in WBC [145]	Increase in tumor inhibition and reduce death rate [68]
Cisplatin	Animal	Prolong survival or increase survival rate [63–66], increase [64, 66, 104, 125, 174] or no effect [146, 147, 175–177] on tumor inhibition, inhibit angiogenesis (decease microvessel density) [104] Influence on inflammatory cytokines (increase in interferons [146], TGF-β [127], increase or decrease in TNF-α [64, 127, 146, 159], IL [64, 127, 146, 159]) and phagocytic function [159] Increase in T cells (CD3+, CD4+, CD8+), NK cells and CD11c+ DC cells [65, 66, 125–127, 146] Increase blood and renocortical SOD, glutathione, GPx and reduce MDA [64, 175–180, 190–192] Reduces damage to liver (reduce ALT, AST) [66], kidney (reduce Scr, urea, BUN, ALP, urine NAG, toxic accumulation of plasma infiltrate, enhance CAT activity, renal structure) [64–66, 174–180, 192], intestines [173] and relieve nausea/vomiting [147, 183, 184] Modulate Bax, Bcl-2, caspase 3 [66, 125], aquaporin [125, 126], VEGF, bFGF [104, 127] expression	Increase in tumor inhibition [113] Increase in CD4+, CD4+/CD8+, decrease in CD8+ T cell [113] Upregulate Fas and caspase 3 expression, downregulate Fasl expression [113] Decrease cisplatin-induced raise in BUN and Scr [166]
	In-vitro	Increase tumor growth inhibition [104–109], apoptosis [122, 123], inhibit angiogenesis [104] Enhance drug sensitivity through the JAK–STAT3 pathway [123]. Reverse resistance [106, 109] Modulate Fas/FasL-mediated apoptosis [107] Induce cell cycle arrest by interfering with HER2/PI3K/Akt pathway [108]. Modification of the expression of TGF-β1 [105], Smad4 [105], VEGF, bFGF [104], HER2 [108], ABCB1 [122], Bax, Bad, Bcl-2, Bcl-xL [107], Akt, p53 [106]	Increase [131] or no effect [119] on tumor cell inhibition Prevent inhibition on normal cells by cisplatin [131] Prevent decrease of SOD and increase in lipid peroxide in normal cells, but opposite effect in cancer cells [131]
Cyclophosphamide	Animal	Increase in tumor inhibition [81, 82, 85–94] and apoptosis [81, 89, 91], suppress metastasis [90] (increase TMSG-1 expression) [91], increase in survival time [81, 82, 163] Relieve BMS, including RBC [87, 150, 156, 163, 164], WBC (NK and T cells) [81, 85, 87, 89, 90, 147–156, 158, 163], platelets [150, 153, 163], hemoglobin [163–165], phagocytic activity, [85, 151, 155, 156, 158, 161, 165] cytokines and antibody production [81, 88–90, 149, 150, 152, 153, 160] Increase antioxidant capacity, SOD, CAT, GPx and reduce MDA to normal level [81, 150] Induce loss of Bcl-2 and Bax translocation, induce release of cytochrome c, increase caspase 3 and 9 activities [81] Protect against liver (Less ALT and AST increase) [86, 164, 165] and intestine damage [173] Inhibit mutation (decrease in micronuclei frequency) [185] Reduce weight loss [85, 90, 92, 160, 163], fatigue and appetite loss [93, 94, 163, 165]	Increase in tumor inhibition [69–71, 112] through enhancement of cytotoxicity of lymphokine-activated killer cells and tumor-infiltrating lymphocytes [70] Reduce metastasis [70, 71] and increase survival [69–71] Less decrease in WBC (B and T cells, NK cells) [69, 166–168], hemoglobin [69] Reduce immunosuppression, increase in antibody production [112] and immunoglobulin [167] Decrease expression of immune negative transcription factors such as Foxp3, PD-1, IL-10 [171], IL-4, GATA-3 and modulate the unbalanced T helper cells [169]
	In-vitro	Increase cytotoxicity to tumor cells and drug sensitivity [103]	Enhance cytotoxicity of drug while itself has no cytotoxic effect [18]
Cytarabine	In-vitro	No studies available	Decrease expression of Bax, Bcl-xL and Bcl-xL/Bax ratio [128]
Docetaxel	Animal	No studies available	Increase tumor inhibition [114, 115, 117], apoptosis [114], reduce metastasis [116] Less decrease in WBC, NK-cell [114], increase CD4+, CD8+ T cells, IL [114] Suppress induced expression of NF-kB and survivin [117]
	In-vitro	No studies available	Enhance anti-tumor effect (dose-dependent) [115, 117, 119], apoptosis [115, 120] and reduce docetaxel-enhanced invasion [115] Suppress induced expression of survivin [117], NF-kB [115, 117, 120], MMP-9 [115]. Inhibit expression of cIAP-1, enhance caspase-3 activation [120]
Doxorubicin	Animal	Prolong survival in additive function [63] Less decrease in WBC and platelet [157] Relieve myocardial and hepatocellular injury through modulation of enzymes (ALT, AST, LDH, CK) and oxidative stress biomarkers (GST, GPx, SOD, CAT) [157, 181, 182]	Less decrease in CD3+, CD4+ T cells, IL-2 and IL-2R expression [129, 130] Increase Bax, decrease Bcl-2 and CDK4 [129, 130]
	In-vitro	Synergistic [15, 124] or no effects [14] on tumor inhibition, reverse resistance [14, 15] Increase apoptosis, decrease Ku80 and enhance reactive oxygen species production [124]	Enhance apoptotic effect [128] Increase expression of Bax, decrease Bcl-xL, Bcl-xL/Bax ratio [128]
Epirubicin	Animal	Less reduction of WBC [145]	No studies available
Etoposide	Animal	No studies available	Increase tumor inhibition and reduce death rate [68]
	In-vitro	No studies available	Enhance apoptotic effect [128] Increase Bid, decrease Bcl-xL expression and Bcl-xL/Bax ratio [128]
Gemcitabine	In-vitro	No studies available	Additive effect in tumor growth inhibition [121]
Mercaptopurine	Animal	No studies available	Increase delayed hypersensitivity reaction [112]
Methotrexate	Animal	Prolong survival in additive function [66] Reduce induced small intestinal damage [162] Dose-related increase in immunoglobulin A and modulate change in oxidative stress marker such as SOD and MDA induced by methotrexate [162]	Reduce immunosuppression (increase delayed hypersensitivity reaction) [112]
Mitomycin	Animal	No significant protection against mitomycin-induced mutation [185]	Increase in survival rate or time [72–74], tumor growth inhibition [73] Dose-related decrease in frequency of sister chromatid exchanges [188] Recover antibody production and delayed-type hypersensitivity [73, 112] Decrease incidence of high mobility cells, increase low mobility cells [72]
	In-vitro	Increase in cytotoxicity to tumor cells [103] Protection against mutation damage [185]	Reduce micronuclei formation in dose-related manner [187]
Paclitaxel	Animal	Increase in tumor inhibition [95–97] and efficacy of paclitaxel [95] Increase let-7 expression [95] Restore antitumorigenic immune cells via inhibiting immune checkpoints [97] Down-regulation of Warburg effect-related proteins to inhibit tumor metabolism [97] Restore gut dysbiosis induced by paclitaxel [97]	No studies available
	In-vitro	Enhance tumor growth inhibition [96, 108, 110] and apoptosis [96], reverse resistance [14] Induce cell cycle arrest by interfering with HER2/PI3K/Akt pathway [108], inhibition of HER2 signaling pathway and downregulate expression of HER2 related proteins [96, 108]	No studies available
Retinoic acid	Animal	Reduce neural tube defects through up-regulating the transcription of CDK4 mRNA and expression of CDK4 and nestin at neural tube epithelia [186]	No studies available
Thioguanine	Animal	Prolong survival in additive function [63]	No studies available
UFT/Tegafur	Animal	No significant increase in efficacy [147] Reduce damage to intestine [173] and less reduction in WBC, but not platelet [147]	Increase in cytotoxicity and inhibit metastasis [111] Reduce immunosuppression (phagocytic activity, antibody production) [111]
Vincristine	In-vitro	Synergistic [110] or no significant effect [14] in tumor inhibition and reverse resistance [14]	No studies available

##### Effect on survival and quality of life

*a. Clinical evidence*

Both Lingzhi and Yunzhi showed survival benefits in clinical studies. In this review, Yunzhi showed more clinical evidence than Lingzhi on increase in the survival rate and prolongation of the survival time. Among the 21 clinical studies on Lingzhi, only three studies measured survival outcomes with two of them reported increase in survival rate [19–21]. The survival rate increased when herbal formulae containing Lingzhi were used together with chemotherapy regimens (including 5-FU, cisplatin, doxorubicin, mitomycin, or vincristine). Notably, 37 of the 56 clinical studies on Yunzhi evaluated survival outcomes, 36 of which used PSK and one used Yunzhi-containing herbal formula. Twenty-nine of the 37 studies showed survival benefits of Yunzhi when co-administered with cytotoxic drugs. Among these Yunzhi–drug combinations, more evidence of survival benefits was reported for combinations with 5-FU, mitomycin, and UFT or Tegafur [22–50], whereas no survival benefits were reported for the combination with leucovorin [51]. Most of the Japanese studies on Yunzhi showed survival benefits of 3 or 5 years in gastric and colorectal cancers, leading to the approval of PSK approved as an adjunct in cancer treatment in Japan.

In terms of quality of life, Lingzhi co-administration with cytotoxic drugs has more clinical evidence than that for Yunzhi. Among the 21 included studies for Lingzhi, 12 of them measured the quality of life of cancer patients, and all of them observed an improvement by the co-administration of Lingzhi with 5-FU, capecitabine, cisplatin, gemcitabine, leucovorin, oxaliplatin, paclitaxel, and vinorelbine. The improvement in the quality of life was mostly observed in lung cancer patients (8 of the 12 studies) [21, 52–59]. Among the included studies, only 5 of the 56 clinical studies on Yunzhi measured the quality of life of cancer patients after the co-administration of Yunzhi with various cytotoxic drugs (5-FU, cisplatin, carboplatin, capecitabine, cyclophosphamide, doxorubicin, etoposide, mitomycin, oxaliplatin, paclitaxel and UFT) and all of them showed improvement [48, 49, 60–62].

Overall, more clinical evidence was reported on the survival benefits for the co-administration of chemotherapy with Yunzhi specifically PSK especially in gastric and colorectal cancer, whereas more evidence was reported on the improvement in the quality of life for the co-administration of chemotherapy with Lingzhi, especially in lung cancer patients.

*b. Preclinical evidence*

Animal studies on the concurrent use of Lingzhi or Yunzhi with cytotoxic drugs also measured the survival outcomes and substantiated the findings of the above-mentioned clinical studies. With regard to Lingzhi, its survival benefits were also observed in animal studies when co-administered with 5-FU, carboplatin, cisplatin, and doxorubicin which also shown benefits in clinical studies [63–66]. With regard to Yunzhi, the survival rate or time of animals increased when it was co-administered with 5-FU, cyclophosphamide, etoposide, and mitomycin, which were consistent with the findings from clinical studies [67–74].

##### Effect on tumor inhibition and disease control rate

*a. Clinical evidence*

Most of the clinical studies identified in our review measured efficacy based on World Health Organization or the Response Evaluation Criteria in Solid Tumor (RECIST) [75]. The co-administration of Lingzhi with various cytotoxic drugs showed an increase in disease control rate or clinical efficacy via the reduction of tumor lesion size; these drugs include 5-FU, capecitabine, cisplatin, doxorubicin, gemcitabine, leucovorin, mitomycin, oxaliplatin, paclitaxel, and vincristine [19–21, 52–56, 59, 76–78]. In addition, co-administration of Lingzhi with 5-FU and cisplatin could further reduce the expressions of microRNA-21 (miR-21), endoglin, transforming growth factor (TGF)-β, and vascular endothelial growth factor (VEGF) [76].

The co-administration of Yunzhi with several cytotoxic drugs (carboplatin, capecitabine, cisplatin, cyclophosphamide, doxorubicin, etoposide, mercaptopurine, oxaliplatin and paclitaxel) showed increase in clinical efficacy [49, 62, 79, 80] and improvements in disease progression or control [48, 50]. Some Yunzhi–drug combinations (5-FU, carboplatin, cisplatin, cyclophosphamide, doxorubicin, etoposide and UFT) also reduced tumor recurrence [25, 38, 42, 44] and metastasis [49]. Lower level of markers for tumor cell division, proliferation and invasion were found when paclitaxel and carboplatin were used with PSP [62].

Notably, most studies showed an enhancement in the cytotoxicity toward tumor cells when cytotoxic drug were used with these two medicinal mushrooms, suggesting no disadvantage for the addition of mushrooms in chemotherapy regimens.

*b. Preclinical and mechanistic evidence*

Our review revealed that the mechanisms of interactions between Lingzhi or Yunzhi and cytotoxic drugs involve multiple targets, including genes, proteins, and signaling pathways, to synergistically increase the tumor-inhibitory effect. Most animal studies support the clinical findings above. For Lingzhi, increase in the tumor inhibition rate was also demonstrated in animal studies [81–98] and in-vitro studies [96–110]. Some animal studies also observed suppression of metastasis by the co-administration of Lingzhi with cyclophosphamide [90, 91]. Regarding Yunzhi, enhancement of the antitumor effects of cytotoxic drugs and reduction in metastasis by Yunzhi have also been demonstrated in animal studies [68–71, 73, 111–117] and in-vitro studies [18, 115, 117–121].

The effects of Lingzhi co-administered with cytotoxic drugs on tumor inhibition were multifaceted in both animal and in-vitro studies. Some combinations exert synergistic effect through induction of cell cycle arrest at different stages or increase the apoptotic rate [81, 89, 91, 96, 99–102, 122–124]. In addition, the enhancement of the antitumor effect was shown to be achieved via the inhibition of tumor cell angiogenesis [104] or increase in the reactive oxygen species production [124]. Various in-vitro studies have shown that Lingzhi could enhance the tumor-inhibitory effect by reversing resistance to cytotoxic drugs [14–16]

Lingzhi co-administration with cytotoxic drugs affects different pathways and cancer targets. In animal and in-vitro studies, various pathways related to cell cycle arrest, apoptosis, and angiogenesis were affected, including the Janus kinase/signal transducer and activator of transcription 3 (JAK–STAT3), the protein kinase B (Akt) [106, 123] and the Human epidermal growth factor receptor 2 (HER2) signaling pathway [96, 108], eventually affecting the apoptotic molecules, for example, by altering B-cell lymphoma (Bcl) and Bcl-2-associated X (Bax) [66, 81, 125], caspase 3 and 8 activation [81, 100, 107, 125], and Fas and Fas Ligand (Fasl) [104]. The expression of other target genes or proteins was also affected, such as aquaporin [125, 126], VEGF and basic fibroblast growth factor (bFGF) [104, 127], and tumor metastasis suppressor gene 1 (TMSG-1) protein [91], all of which increased tumor inhibition. Notably, a recent study found that such synergistic effect may be due to the inhibition of tumor metabolism via down-regulating Warburg-related proteins and restoration of the gut dysbiosis induced by paclitaxel [97]. Regarding the increasing evidence of microbiota involvement in chemotherapy outcomes, it is worth looking into interactions with other cytotoxic drugs on gut microbiome level.

For Yunzhi, both PSK and PSP were reported to further enhance the apoptotic activity when co-administered with docetaxel, doxorubicin and etoposide [114, 115, 120, 128]. Similar to cytotoxic drugs, Yunzhi and its components could also act on the (a) expression of cell cycle arrest and apoptosis related genes and proteins, including Fas and Fasl [113]; Bax, Bcl-2, and BH3 interacting-domain death agonist (Bid) [128–130]; nuclear factor-κB (NF-kB) [115, 117, 120]; cellular inhibitor of apoptosis protein 1 (cIAP-1) [120] and caspase-3 [113, 120] and (b) targets related to angiogenesis and cell invasion, including matrix metallopeptidase 9 (MMP-9) [115], leading to synergistic effect when they are used together.

Yunzhi could also enhance the cytotoxicity of anticancer drugs via other mechanisms, such as direct increase in the activity of cytotoxic and tumor-infiltrating lymphocytes, decrease in the activity of suppressor T cells [38, 70], and decrease and increase in superoxide dismutase (SOD) and lipid peroxide levels, respectively, in cancer cells [131]. In addition, PSK was shown to enhance the antitumor effect of 5-FU and Tegafur by downregulating the expression of dihydropyrimidine dehydrogenase messenger RNA (mRNA) [118], as well as docetaxel via the suppression of survivin expression activated by docetaxel [117].

##### Interaction on immune system

*a. Clinical evidence*

In clinical studies, Lingzhi co-administration with cytotoxic drugs generally relieved bone marrow suppression. The combinations alleviated the decrease in white blood cells (WBCs), including lymphocytes and neutrophils, caused by chemotherapy alone (5-FU, capecitabine, cisplatin, doxorubicin, gemcitabine, leucovorin, mitomycin, oxaliplatin, paclitaxel, and vinorelbine). The number of other blood cells, including red blood cells (RBCs), hemoglobin, and platelets was also increased by the combinations compared with chemotherapy alone [19–21, 52–57, 59, 77, 78, 132–134]. Specifically, some combinations alleviated the decrease in or induced an increase in some T cells (CD3+, CD4+, CD8+), CD4+/CD8+ ratio and natural killer (NK) cells [19, 20, 52–55, 59, 76–78, 132–134].

Co-administrations of Yunzhi with various cytotoxic drugs (capecitabine, mercaptopurine, oxaliplatin, and Tegafur) were also shown to reduce immunosuppression in clinical studies [47, 48, 50, 135]. Studies have shown a reversal of the decrease in WBCs, in particular, improvement of the increase in or alleviation of the decrease in T cells (CD3+, CD4+, CD8+), increase in the CD4+/CD8+ ratio, NK cell activity, and subsequent the production of immunoglobulins and interleukins (IL) [23, 38, 49, 60, 62, 68, 79, 80, 136–144]. Inhibition on other blood cells, such as platelets and hemoglobin by cytotoxic drugs were also alleviated by the co-administered Yunzhi [80, 136–140, 144].

*b. Preclinical and mechanistic studies*

Animal studies also showed alleviation in the WBCs decrease with the co-administration of Lingzhi and different cytotoxic drugs, particularly in T cells and NK cells [65, 66, 81, 83–85, 87, 89, 90, 125–127, 145–158, 163]. In addition, phagocytic activity and production of cytokines, including immunoglobulins, interferons (IFN), IL, and TGF, were shown to be restored [64, 81, 85, 88–90, 127, 146, 149–151, 153, 155, 156, 158–162, 165]. Suppression in the number of RBCs, platelets, and in hemoglobin was also found to be alleviated in animal studies [83, 84, 87, 150, 153, 156, 157, 163–165].

The co-administration of Yunzhi with cytotoxic drugs showed similar effects on immune function in animal studies as those observed in clinical studies. Immunosuppression was reduced, including alleviation of the decrease in WBCs, particularly B cell and T cells (CD3+ and CD4+); increase in the CD4+/CD8+ ratio, NK cells and phagocytic activity; and subsequent production of interleukins, immunoglobulins, and antibodies [69, 73, 111–114, 129, 130, 148, 166–168]. Inhibition on other blood cells, including hemoglobin, also was alleviated [69].

A complete understanding of the mechanisms and factors underlying the effects of the interaction of Lingzhi or Yunzhi with cytotoxic drugs on the immune system is still obscure. Multiple mechanisms may be involved, including upregulation of transcription factors such as T-box protein expressed in T cells (T-bet) and GATA-3, which modulate the T helper cell balance [169]; downregulation of Bax expression to reduce caspase 3 activity and T cell apoptosis [170]; and reduction of the expression of negative regulators/transcription factors of immune responses, including Forkhead box protein 3 (Foxp3) and programmed death 1 (PD-1) [171].

##### Alleviation of the other adverse effects of cytotoxic drugs

*a. Clinical evidence*

The co-administration of Lingzhi with some cytotoxic drugs (5-FU, capecitabine, cisplatin, doxorubicin, gemcitabine, leucovorin, mitomycin, oxaliplatin, and paclitaxel) was shown to reduce common adverse effects, such as reduction of gastrointestinal discomfort (nausea, vomiting, constipation, and diarrhea) and stomatitis [19, 53, 56, 59, 77, 78]. Specific adverse effect, such as nephrotoxicity [56] was also shown to be ameliorated.

In clinical studies on Yunzhi, the use of PSK and PSP with cytotoxic drugs (5-FU, capecitabine, mitomycin, oxaliplatin, and UFT) ameliorated the general adverse effects, such as gastrointestinal discomfort (nausea and vomiting, diarrhea, and constipation) [48, 61, 136], mucositis [48], and fatigue [136]. Specific adverse effects of cytotoxic drugs, including peripheral neuropathy [143], mutations [172], and hand-foot syndrome [48], were also reduced.

*b. Preclinical and mechanistic evidence*

The Lingzhi and cytotoxic drug combinations were also found to relieve some adverse effects in animal studies; for example, they reduced damage to different organs, including the intestine [162, 173], the kidney [64–66, 174–180], the liver [66, 86, 157, 164, 165], and the heart [157, 181, 182]; and their adverse effects, such as nausea and vomiting [147, 183, 184], mutation [185], and neural tube defects [186].

Some studies have evaluated the mechanism underlying the protective effects of the mushroom–drug combinations against chemotherapy-induced side effects. For example, the mechanism underlying the reduction of doxorubicin-induced myocardial damage was found to be via the modulation of cardiac enzymes and reduction of the oxidative stress (such as increase in glutathione S-transferase (GST) and glutathione peroxidase (GPx) levels) in myocardial cells by Lingzhi or Ganoderma polysaccharides [181, 182]. Similarly, Lingzhi and its polysaccharides likely increased renocortical antioxidant levels and relieved the oxidative stress, thereby ameliorating cisplatin-induced renal damage. Furthermore, Lingzhi reduced the occurrence of neural tube defects caused by retinoic acid by upregulating the transcription of cyclin-dependent kinase 4 (CDK4) mRNA and the expression of CDK4 and nestin in neural epithelia [186].

For Yunzhi animal studies, cisplatin-induced renal damage was reduced by decreasing blood urea nitrogen (BUN) and serum creatinine (Scr) levels [166]. In both animal and in-vitro studies, PSK reduced the mutagenicity of mitomycin by decreasing the frequency of sister chromatid exchange and micronuclei formation [187, 188].

In summary, the aforementioned outcomes suggest that medicinal mushrooms have great potential to ameliorate the adverse effects of chemotherapeutic drugs in clinical settings.

##### Other effects in pre-clinical studies

Several chemotherapy drugs are known to cause oxidative stress which damage tumor cells but concomitantly damage normal cells and produce adverse effects [189]. The co-administration of Lingzhi with cytotoxic drugs, including cisplatin, cyclophosphamide, doxorubicin, and methotrexate increased the total antioxidant capacity and levels of different antioxidant enzymes, including superoxide dismutase (SOD), glutathione, GPx, and catalase, and decreased malondialdehyde levels, thereby reducing the oxidative stress [64, 81, 150, 157, 162, 175–182, 190–192]. In an in-vitro study, the co-administration of Yunzhi with cisplatin prevented the decrease in SOD activity by mimicking it to protect normal healthy cells while selectively promoting the decrease in SOD activity and increase in lipid peroxide levels in cancer cells, both of which synergistically increased tumor inhibition while leaving normal cells unaffected [131]. However, these findings have not yet been substantiated in clinical settings.

### Undesirable interaction outcome

Overall, there is no reported undesirable herb-drug interaction between Lingzhi or Yunzhi and cytotoxic drugs in our included studies. Although interaction between PSP and cyclophosphamide may potentially increase the systemic exposure of cyclophosphamide, it is not known whether it could lead to significant changes in clinical outcomes [193]. Since our findings indicated that Lingzhi/Yunzhi and their components may potentially affect pharmacokinetics of anticancer drug, further clinical pharmacokinetic studies are warranted to investigate the necessity for dose adjustment of anticancer drug in clinical practice.

### Quality of clinical studies

The clinical studies on the concurrent use of Lingzhi or Yunzhi with cytotoxic drugs were rated using the Jadad scale. Overall, most of the clinical studies on the combined use of mushrooms and cytotoxic drugs were not of high quality, scoring mostly 0–3 points. Most of them did not adopt blinding measures, probably because it is not feasible to blind chemotherapy regimens and complex herbal therapies. In addition, many studies lacked comprehensive description of withdrawal and dropouts. Only half of the randomized studies provided details on their randomization methods. Similar to the findings from others on clinical trials with Chinese medicine [194], the trials involved in the current review were also with poor clinical trial design and insufficient reporting of studies. Although the SPIRIT 2013 and CONSORT 2010 guideline have been published with intent to improve the design and reporting of randomized controlled trials, they may not be completely applied to the trials of Chinese medicine formulas. Thus, a CONSORT 2010 extension and SPIRIT-TCM extension have been established in 2017 and 2018 respectively to meet the unique characteristics of Chinese medicine [195, 196], which could serve as the guidance for future clinical studies involved Chinese medicines.

## Conclusion and implication for future study

The combination of Lingzhi and Yunzhi with cytotoxic anti-cancer drugs showed great potential in offering beneficial effects in clinical settings with no undesirable interactions reported so far. The survival benefits can be increased, especially for Lingzhi and the quality of life of cancer patients can be enhanced especially for Yunzhi. Both Lingzhi and Yunzhi showed synergistic effect on tumor inhibition with chemotherapy, reducing immunosuppression and alleviating general and specific chemotherapy related side effects.

Despite a relatively large number of clinical studies conducted, the quality of clinical studies included in this review remained not high, especially with lack of blinding and description of all withdrawals and dropouts. Trials based on guidance from CONSORT 2010 extension and SPIRIT-TCM extension are highly recommended in the future to ensure the higher quality of clinical trial on Chinese medicines. Pharmacokinetic effects of Lingzhi or Yunzhi on cytotoxic drugs were inconclusive. There was currently significantly lack of studies on pharmacokinetic herb-drug interactions as revealed above. Considering the narrow therapeutic index of many cytotoxic drugs, more clinical pharmacokinetic studies are warranted for their safe and effective use.

In recent years, there are revolutionary advancements in the oncology world that sees many new breakthrough treatments, including targeted therapies, immunotherapy as well as gene therapy. Future studies should identify potential synergistic and harmful interaction between Chinese Medicine and these advanced cancer therapies. With increasing number of studies on combined usage of Chinese and western medicine, future studies can harness modern techniques such as bioinformatics and data mining to identify patterns of herb–herb and drug-herb combinations, especially those show the strongest evidence for tumor inhibition. Moreover, considering the large population utilizing Chinese Medicine in Asian countries and increasing prevalence in Europe and US, future pharmacoepidemiology studies can be conducted to observe the clinical impact of integrative medicine among cancer patients in a real word setting, in particular long-term and delayed effect that cannot be revealed in ordinary clinical trial settings.

## Supplementary information

**Additional file 1. Table S1.** Keywords for cytotoxic drugs in the current database search. **Table S2.** Characteristics of included studies. **Table S3.** Detailed information of the included clinical studies. **Table S4.** Detailed information of the included pre-clinical studies. **Figure S1.** Distribution of pre-clinical and clinical studies for combination use of cytotoxic drugs with Lingzhi and Yunzhi.

## Data Availability

Not applicable.

## Abbreviations

5-FU: 5-Fluorouracil
ABCB1: ATP binding cassette subfamily B member 1
Akt: Protein kinase B
ALP: Alkaline phosphatase
ALT: Alanine transaminase
AST: Aspartate aminotransferase
Bax: Bcl-2-associated X
Bcl: B-cell lymphoma
bFGF: Basic fibroblast growth factor
Bid: BH3 interacting-domain death agonist
BMS: Bone marrow suppression
BUN: Blood urea nitrogen
CDK4: Cyclin-dependent kinase 4
CEA: Carcinoembryonic antigen
cIAP-1: Cellular Inhibitor of Apoptosis Protein 1
CK: Creatine kinase
DC: Dendritic cells
Fasl: Fas ligand
Foxp3: Forkhead box P3
GPx: Glutathione peroxidase
GST: Glutathione *S*-transferase
HER2: Human epidermal growth factor receptor 2
IFN: Interferon
IL: Interleukin
JAK–STAT3: Janus kinase/signal transducer and activator of transcription 3
LBT: Lymphocyte blastogenic transformation
LDH: Lactate dehydrogenase
MDA: Malondialdehyde
miR-21: microRNA 21
MMP-9: Matrix metallopeptidase 9
mRNA: Messenger RNA
NAG: *N*-acetyl-beta-d-glucosaminidase
NF-kB: Nuclear factor-κB
NK: Natural killer
PD-1: Programmed death 1
PI3K: Phosphoinositide 3-kinase
PSK: Polysaccharide K
PSP: Polysaccharide peptide
p53: Tumor protein p53
RBC: Red blood cells
Scr: Serum creatinine
Smad4: Mothers against decapentaplegic homolog 4
sMICA: Soluble major histocompatibility complex class I-related chain A
SOD: Superoxide dismutase
T-bet: T-box protein expressed in T cells
TGF: Transforming growth factor
TMSG-1: Tumor metastasis suppressor gene 1
UFT: Tegafur/uracil
VEGF: Vascular endothelial growth factor
WBC: White blood cells
