# Supplementary material for: Herb–drug interactions between the medicinal mushrooms Lingzhi and Yunzhi and cytotoxic anticancer drugs: a systematic review
Source: Chin Med. 2020 Jul 25;15:75. doi: 10.1186/s13020-020-00356-4 (PMC7382813; doi:10.1186/s13020-020-00356-4)
Supplement: Supplementary file 1 — Additional file 1. Table S1. Keywords for cytotoxic drugs in the current database search. Table S2. Characteristics of included studies. Table S3. Detailed information of the included clinical studies. Table S4. Detailed information of the included pre-clinical studies. Figure S1. Distribution of pre-clinical and clinical studies for combination use of cytotoxic drugs with Lingzhi and Yunzhi. [file 13020_2020_356_MOESM1_ESM.pdf]

Additional File 1

**Table S1**	**Keywords for cytotoxic drugs in the current database search**
**Table S2**	**Characteristics of included studies**
**Table S3**	**Detailed information of the included clinical studies**
**Table S4**	**Detailed information of the included pre-clinical studies**
**Figure S1**	**Distribution of pre-clinical and clinical studies for combination use of cytotoxic drugs with Lingzhi and Yunzhi.**

Table S1 Keywords for cytotoxic drugs in the current database search

Generic Name	Brand Name (US, Japan, HK)	Chinese Name (Chinese Pharmacopoeia 2015)
Asparaginase, crisantaspase	Leunase, Erwinaze	門冬酰胺
Bleomycin	Bleocin, Blenoxane	博來霉素，平陽霉素
Busulphan, Busulfan	Myleran, Busulfex	白消安
Calcium Folinate, Leucovorin, Folinic acid	Fusilev	阿葉酸鈣，甲酰四氫葉酸，
Capecitabine	Xeloda	卡培他濱
Carboplatin	Paraplatin	卡鉑
Carmustine	BiCNU, Gliadel	卡莫司汀
Chlorambucil	Leukeran	苯丁酸氮芥
Clofarabine	Clolar, Evoltra	氯法拉濱
Cisplatin	Randa, Platinol	順鉑
Cyclophosphamide	Endoxan, Neosar, Cytoxan	環磷酰胺
Cytarabine	Alexan, Cytosar, Arabitin, Cyclocide, Starasid, Depocyt, Vyxeos	阿糖胞苷
Dacarbazine	DTI	達卡巴嗪
Daunorubicin	Daunoxome, Cerubidin	柔紅霉素
Docetaxel	Docefrex, Taxotere	多西他奇，多西他賽，多西紫杉醇
Doxorubicin	Adriacin, Adriamycin	多柔比星，阿霉素
Epirubicin	Pharmorubicin, Episindan, Farmorubicin, Ellence	表阿霉素，表柔比星
Etoposide	Lastet, Sintopozid, Vepesid, Etopophos, Toposar	足葉乙甙，依托泊苷
Fludarabine	Fludara, Oforta	氟達拉賓
Fluorouracil, 5-FU	Adrucil	氟尿嘧啶
Gemcitabine	Gemzar, Gitrabin	吉西他濱
Hydroxyurea	Droxia, Hydrea, Siklos	羥基脲
Idarubicin	Idamycin, Zavedos	伊達比星/去甲氧柔紅黴素
Ifosfamide	Ifex, Holoxan, Ifomide	異環磷酰胺
Melphalan	Alkeran, Evomela	馬法蘭
Mercaptopurine	Purinethol, Purixan	巰基嘌呤，巰嘌呤
Mesna	Mesnex, Uromitexan	美司鈉
Methotrexate	Otrexup, Rasuvo, Xatmep, Rheumatrex, Trexall	甲氨蝶呤，氨甲蝶呤，甲氨喋呤
Mitomycin	Mytomycin, Mutamycin	絲裂霉素
Mitoxantrone	Santrone, Novantrone	米托蒽醌
Oxaliplatin	Eloxatin, Elplat	奥沙利鉑
Paclitaxel	Abraxane, Taxol, Anzatax, Ebetaxel, Sindaxel	紫杉醇
Pemetrexed	Alimta	培美曲塞
Teniposide	Vumon	替尼泊甙
Thioguanine	Tioguanine, Tabloid, Lanvis	硫鳥嘌呤
Tretinoin	Vesanoid	維甲酸
Tegafur-Uracil, Tegafur, Uracil, UFT	-	替加氟，尿嘧啶
Vinblastine	Velban, Velbastine	長春堿
Vincristine	Marqibo, Oncovin, Vincrex, Vincasar, Kyocristine, Vinrasine, Vinca alkaloids	長春新堿
Vinorelbine	Navelbine	長春瑞濱

Table S2 Characteristics of included studies

	Lingzhi	Yunzhi
**Languages**		
Chinese	89 (74.2%)	28 (29.8%)
English	29 (24.2%)	61 (64.9%)
Japanese	2 (1.67%)	5 (5.32%)
**Study type***		
Clinical	21 (17.5%)	56 (59.6%)
Animal	76 (63.3%)	32 (34.0%)
In-vitro	27 (22.5%)	10 (10.6%)
**Country of origin of clinical studies**		
China	21 (100%)	14 (25.0%)
Japanese	0 (0%)	42 (75.0%)
**Cancer Type in clinical studies***		
Lung	17 (81.0%)	5 (8.93%)
Colorectal	5 (23.8%)	14 (25.0%)
Gastric	4 (19.0%)	24 (42.9%)
Cervical	3 (14.3%)	1 (1.79%)
Esophageal	3 (14.3%)	3 (5.36%)
Bladder	2 (9.52%)	0 (0%)
Liver	2 (9.52%)	2 (3.57%)
Breast	0 (0%)	4 (7.14%)
Ovarian	0 (0%)	2 (3.57%)
Head and neck	0 (0%)	1 (1.79%)
**Type of medicinal mushroom preparation used in clinical studies**		
Multi-herbal formula	16 (76.2%)	1 (1.79%)
Single herb	5 (23.8%)	1 (1.79%)
Herbal components	0 (0%)	54 (96.4%)
**Type of medicinal mushroom preparation used in pre-clinical studies***		
Multi-herbal formula	13 (13.1%)	3 (7.69%)
Single herb	37 (37.4%)	0 (0%)
Herbal components	49 (49.5%)	39 (100%)
**Jadad score of clinical studies**		
5	1 (4.76%)	0 (0%)
4	1 (4.76%)	1 (1.79%)
3	5 (23.8%)	8 (14.3%)
2	3 (14.3%)	7 (12.5%)
1	10 (47.6%)	15 (26.8%)
0	1 (4.76%)	25 (44.6%)

*The percentage in total may be more than 100% since there are multiple study types/ cancer types/ forms of medicinal mushroom used in pre-clinical studies in single literature.

Table S3 Detailed information of the included clinical studies

Author (year)	Sample size	Cancer type	Treatment (chemotherapy regimen with mushrooms)	Control (chemotherapy-only/ mushroom-only regimen)
**Lingzhi**				
Chen et al. (2015)	83	Lung	Paclitaxel 135 mg/m^2 IV on day 1, Cisplatin 75 mg/m^2 IV on day 1-3/1-4 of 21-day cycle + Yang Zheng Xiao Zhi Capsule (including Lingzhi) 4 capsules PO three times daily	Paclitaxel 135 mg/m^2 IV on day 1, Cisplatin 75 mg/m^2 IV on day 1-3/1-4 of 21-day cycle
Hu & Gan. (2013)	60	Lung	Gemcitabine 1000 mg/m^2 IV day 1 and 8, Cisplatin 60 mg/m^2 IV day 3-5 of 21-day cycle + Bailonglingsha Decoction (include Lingzhi 10 g) 200 ml PO daily	Gemcitabine 1000 mg/m^2 IV day 1 and 8, Cisplatin 60 mg/m^2 IV day 3-5 of 21-day cycle
Jing et al. (2007)	114	Esophageal, gastric, colon	Cisplatin 30 mg/m^2 IV, 5-FU 500 mg/m^2, Calcium folinate 100 mg IV on day 1-5 of 21-day cycle + Bai Long capsule 0.5 g 3 cap PO three times daily/ oral solution 50 ml (include Lingzhi) PO three times daily	Cisplatin 30 mg/m^2 IV, 5-FU 500 mg/m^2, Calcium folinate 100 mg IV on day 1-5 of 21-day cycle
Li & Zhang. (1998)	56	Lung	Cisplatin 40 mg IV, Vindesine IV + Lao Jun Xian oral solution 20ml (include Lingzhi) PO three times daily	Cisplatin 40 mg IV, Vindesine IV
Lin et al. (2004)	114	Gastric, esophageal, lung, liver, colon, cervical, bladder	5-FU 300 mg/m^2 IV twice weekly, Doxorubicin 30mg/m^2 IV weekly, Mitomycin 3 mg/m^2 IV bolus weekly + Lingzhi capsule 4 cap PO four times daily	5-FU 300 mg/m^2 IV twice weekly, Doxorubicin 30mg/m^2 IV weekly, Mitomycin 3 mg/m^2 IV bolus weekly
Liu (2011)	30	Colorectal	Oxaliplatin 100 mg/m^2 IV on day 1, Leucovorin 400mg/m^2 IV on day 1, 5-FU 400mg/m^2 IV bolus on day 1, 2.4-3.0 g/m^2 IV for 46h + Yi Qi Yang Yin granules (include Lingzhi 30 g) PO twice daily	Oxaliplatin 100 mg/m^2 IV on day 1, Leucovorin 400mg/m^2 IV on day 1, 5-FU 400mg/m^2 IV bolus on day 1, 2.4-3.0 g/m^2 IV for 46h
Lu. (2013)	120	Gastric	Paclitaxel 75 mg/m^2 IV, Oxaliplatin 85 mg/m^2 IV, Calcium folinate 200 mg/m^2 IV, 5-FU 400-600 mg/m^2 IV+ Herbal Formula (include Lingzhi 10 g) PO daily	Paclitaxel 75 mg/m^2 IV, Oxaliplatin 85 mg/m^2 IV, Calcium folinate 200 mg/m^2 IV, 5-FU 400-600 mg/m^2 IV
Wang et al. (2014)	58	Lung	Paclitaxel 135-175 mg/m^2 IV on day 1, Cisplatin 20-25 mg/m^2 IV on day 1-3 of 21-day cycle + Cracked Lingzhi Spore powder 1 g PO three times daily	Paclitaxel 135-175 mg/m^2 IV on day 1, Cisplatin 20-25 mg/m^2 IV on day 1-3 of 21-day cycle
Wang et al. (2014)	64	Colorectal	Oxaliplatin 100-130 mg/m^2 IV on day 1, Capecitabine 1000 mg/m^2 PO twice daily on day 1-14 of 21-day cycle + Cracked Lingzhi Spore powder 10g PO three times daily	Oxaliplatin 100-130 mg/m^2 IV on day 1, Capecitabine 1000 mg/m^2 PO twice daily on day 1-14 of 21-day cycle
Wang et al. (2016)	134	Lung	Paclitaxel 135 mg/m^2 on day 1, Cisplatin 75 mg/m^2 on day 1 of 21-day cycle + Compound Lingzhi Spore capsule (Cracked Lingzhi Spore + Nu Zhen Zi) 4 g PO three times daily	Paclitaxel 135 mg/m^2 on day 1, Cisplatin 75 mg/m^2 on day 1 of 21-day cycle
Xie. (2016)	120	Lung	Paclitaxel 175 mg/m^2 IV on day 1, Cisplatin 25 mg/m^2 IV on day 1-3 of 21-day cycle + Jia Wei Shen Qi Decoction (include Lingzhi 6 g) 400 ml PO daily	Paclitaxel 175 mg/m^2 IV on day 1, Cisplatin 25 mg/m^2 IV on day 1-3 of 21-day cycle
Xu & Zhang. (2008)	30	Lung	Paclitaxel 75 mg/m^2 IV on day 1 and 8, Cisplatin 20 mg/m^2 IV on day 1-3 of 28-day cycle; Gemcitabine 1 g/m^2 IV on day 1 and 8, Cisplatin 20 mg/m^2 IV on day 1-3 of 28-day cycle + Bu Xu Hua Du Formula (including Lingzhi) PO, Jin Shui Bao capsules 4 cap PO three times daily	Paclitaxel 75 mg/m^2 IV on day 1 and 8, Cisplatin 20 mg/m^2 IV on day 1-3 of 28-day cycle; Gemcitabine 1 g/m^2 IV on day 1 and 8, Cisplatin 20 mg/m^2 IV on day 1-3 of 28-day cycle

Table S3 Detailed information of the included clinical studies (con't)

Author (year)	Sample size	Cancer type	Treatment (chemotherapy regimen with mushrooms)	Control (chemotherapy-only/ mushroom-only regimen)
**Lingzhi (con't)**				
Xu et al. (2011)	121	Lung	Cisplatin 70-80 mg/m^2 IV on day 1-3, Vinorelbine 25-30 mg/m^2 IV on day 1-8 + Kangliuzengxiao decoction (include Lingzhi 24 g) 150 ml PO twice daily during chemo, Feiyanning decoction (include Lingzhi 30 g) 150 ml PO twice daily after chemo	Cisplatin 70-80 mg/m^2 IV on day 1-3, Vinorelbine 25-30 mg/m^2 IV on day 1-8
Yan. (2017)	162	Cervical	Cisplatin 75 mg/m^2 IV on day 1, 5-FU 750 mg/m^2 IV on day 1-5 of 21-day cycle + Lingzhi capsule 0.54 g PO three times daily	Cisplatin 75 mg/m^2 IV on day 1, 5-FU 750 mg/m^2 IV on day 1-5 of 21-day cycle
Zhang & Chang. (2002)	71	Lung	Mitomycin 6-8 mg/m^2 IV on day 1, Vincristine 3-4 mg/m^2 IV on day 1-8, Cisplatin 70-80 mg/m^2 IV on day 1 + Shen Long Jin Ling Pills (including Lingzhi) 1-2 capsules PO three times daily	Mitomycin 6-8 mg/m^2 IV on day 1, Vincristine 3-4 mg/m^2 IV on day 1-8, Cisplatin 70-80 mg/m^2 IV on day 1
Zhang & Deng. (2007)	84	Lung	Doxorubicin 50 mg IA, Cisplatin 60 mg IA, Mitomycin 10 mg IA + Yan Shu injection (including Lingzhi) 30ml/d IV	Doxorubicin 50 mg IA, Cisplatin 60 mg IA, Mitomycin 10 mg IA
Zhang et al. (2006)	36	Colorectal	Capecitabine 1000 mg/m^2 twice daily on day 1-14, Oxaliplatin 135 mg/m^2 IV on day 1 of 21-day cycle, + Lingzhi Capsules PO	Capecitabine 1000 mg/m^2 twice daily on day 1-14, Oxaliplatin 135 mg/m^2 IV on day 1 of 21-day cycle
Zhang et al. (2010)	60	Lung	Paclitaxel 75 mg/m^2 IV on day 1 and 8, Cisplatin 20 mg/m^2 IV on day 1-5 of 28-day cycle; Gemcitabine 1 g/m^2 IV on day 1 and 8, Cisplatin 20 mg/m^2 IV on day 1-5 of 28-day cycle + Bu Xu Hua Du Formula (include Lingzhi) PO	Paclitaxel 75 mg/m^2 IV on day 1 and 8, Cisplatin 20 mg/m^2 IV on day 1-5; Gemcitabine 1 g/m^2 IV on day 1 and 8, Cisplatin 20 mg/m^2 IV on day 1-5
Zhao & He. (2008)	40	Lung	Paclitaxel 135-175 mg/m^2 IV on day 1, Cisplatin 25 mg/m^2 IV on day 2-4 of 21/28-day cycle; Gemcitabine 1 g/m^2 IV on day 1 and 8, Cisplatin 25 mg/m^2 IV on day 2-4 of 21/28-day cycle + Compound Lingzhi Spore Capsule 4 cap PO three times daily	Paclitaxel 135-175 mg/m^2 IV on day 1, Cisplatin 25 mg/m^2 IV on day 2-4 of 21/28-day cycle; Gemcitabine 1 g/m^2 IV on day 1 and 8, Cisplatin 25 mg/m^2 IV on day 2-4 of 21/28-day cycle
Zhao et al. (2015)	59	Lung	Paclitaxel 135 mg/m^2/d IV, Cisplatin 25 mg/m^2/d IV + Compound Lingzhi Spore capsule (Cracked Lingzhi Spore + Nu Zhen Zi) 4 g PO three times daily	Paclitaxel 135 mg/m^2/d IV, Cisplatin 25 mg/m^2/d IV
Zhou. (1997)	104	Gastric, esophageal, lung, liver, colon, cervical, bladder	5-FU 300 mg/m^2 IV twice weekly, Doxorubicin 30 mg/m^2 IV weekly on week 1-4, Mitomycin 3 mg/m^2 IV weekly + Lao Jun Xian oral solution (include Lingzhi) 10 ml PO three times daily	5-FU 300 mg/m^2 IV twice weekly, Doxorubicin 30 mg/m^2 IV weekly on week 1-4, Mitomycin 3 mg/m^2 IV weekly
**Yunzhi**				
Akagi & Baba (2010)	21	Gastric	UFT 300 mg/d PO + PSK 3g/d PO	UFT 300 mg/d PO
Anai et al. (1991)	10	Gastric	Tegafur 600 mg PO daily + PSK 3g PO daily	N/A
E et al. (1995)	60	Lung	Mitomycin 10 mg/m^2 IV, Vincristine 1 mg/m^2 IV, Cisplatin 60-90 mg/m^2 IV every 3-4 weeks + PSP capsules 3 cap PO three times daily	Mitomycin 10 mg/m^2 IV, Vincristine 1 mg/m^2 IV, Cisplatin 60-90 mg/m^2 IV every 3-4 weeks
Gao et al. (1999)	50	Lung	Cisplatin 100 mg/m^2 IV, Mitomycin 6 mg/m^2 IV, Vindesine 5mg IV on day 1 every 4 weeks + PSP 1.2 g PO three times daily	Cisplatin 100 mg/m^2 IV, Mitomycin 6 mg/m^2 IV, Vindesine 5mg IV on day 1 every 4 weeks

Table S3 Detailed information of the included clinical studies (con't)

Author (year)	Sample size	Cancer type	Treatment (chemotherapy regimen with mushrooms)	Control (chemotherapy-only/ mushroom-only regimen)
**Yunzhi (con't)**				
Han et al. (2019)	88	Esophageal	Paclitaxel 175 mg/m^2 IV, Carboplatin (AUC=5) IV once every 4 weeks + PSP capsules 3 cap PO three times daily	Paclitaxel 175 mg/m^2 IV, Carboplatin (AUC=5) IV once every 4 weeks
Hattori et al. (1979)	110	Gastric	Tegafur 1 g rectal for one month then 600 mg PO daily + PSK 6 g/d PO	Tegafur 1 g rectal for one month then 600 mg PO daily or PSK 6 g/d PO
Hattori et al. (1990)	7637	Gastric	Tegafur 600 mg/d PO till 150 g + PSK 3 g/d PO until 750g	Tegafur 600 mg PO daily till 150 g
Hattori et al. (1992)	914	Breast	Mitomycin 13 mg/m^2 IV on operation day, Tegafur 600 mg PO daily + PSK 3g PO daily / Tamoxifen 20 mg PO daily	Mitomycin 13 mg/m^2 IV on operation day, Tegafur 600 mg PO daily
Hsu et al. (2017)	918	Gastric	UFT PO or 5-FU IV + PSK 1 g PO three times daily	UFT PO or 5-FU IV
Huang & Cai. (2000)	48	Lung	Etoposide 100mg/d IV on day 1-5, Cisplatin 60 mg/m^2 IV on day 3 every three weeks + PSP capsules 3 cap PO three times daily	Etoposide 100mg/d IV on day 1-5, Cisplatin 60 mg/m^2 IV on day 3 every three weeks
Ishibashi et al. (2008)	55	Colorectal	UFT PO, UFT/Leucovorin PO, 5-FU/Leucovorin IV + PSK PO	UFT PO, UFT/Leucovorin PO, 5-FU/Leucovorin IV
Ito et al. (2004)	446	Colorectal	PSK 3 g PO daily + 5-FU 200 mg PO daily	5-FU 200 mg PO daily
Kaibara et al. (1976)	66	Gastric	Mitomycin 0.4 mg/kg IA during operation + Miromycin 0.4 mg/kg IV 4 weeks post-op, then 0.2 mg/kg every 3 month, Tegafur 800 mg/d PO + PSK 3 g/d PO	Mitomycin 0.4 mg/kg IA during operation only
Kano et al. (1981)	157	Gastric	Mitomycin 20 mg IV intraoperatively and 10 mg IV every 3 months, Tegafur 600 mg PO daily + PSK 3 g PO daily	Mitomycin 20 mg IV intraoperatively
Kano et al. (1982)	324	Gastric	Mitomycin 20 mg IV during operation + Mitomycin 10 mg IV post-op every 3 months, Tegafur 600mg/d PO + PSK 3 g/d PO	Mitomycin 20 mg IV during operation
Kano et al. (1983)	285	Gastric	Mitomycin 20 mg IV intraoperatively and 10 mg IV every 3 months, Tegafur 600 mg PO daily + PSK 3 g PO daily	Mitomycin 20 mg IV intraoperatively
Katoh. (1998)	15	Lung	Etoposide 25mg IV twice daily, Carboplatin (target AUC: 3 mg/ml·min) IV + PSK 3g daily	Etoposide 25mg IV twice daily, Carboplatin (target AUC: 3 mg/ml·min) IV
Kikuchi wt al. (1988)	77	Ovarian	Cisplatin 30 mg/m^2/d IV on day 1, Doxorubicin 7 mg/m^2/d IV on day 1-5, Cyclophosphamide 140 mg/m^2/d IV on day 1-5 + PSK 3g PO daily	Cisplatin 30 mg/m^2/d IV on day 1, Doxorubicin 7 mg/m^2/d IV on day 1-5, Cyclophosphamide 140 mg/m^2/d IV on day 1-5
Kodama et al. (1982)	487	Gastric	Mitomycin 0.4mg/kg IV bolus intraoperatively, then 0.2 mg/kg every 3 months, Tegafur 600-800 mg PO daily + PSK 3 g PO daily from the 7th to 10th day post-op	Mitomycin 0.4mg/kg IV bolus intraoperatively
Kono et al. (2008)	20	Gastric	S-1 80 mg/m^2/d PO + PSK 3g/d PO	S-1 80 mg/m^2/d PO
Kuroda et al. (1998)	357	Gastric	Mitomycin C ± Doxorubicin, Tegafur or Tegafur only + PSK PO (total dose >180g)	Mitomycin C ± Doxorubicin, Tegafur or Tegafur only + PSK PO (average dose of 92.4g)

Table S3 Detailed information of the included clinical studies (con't)

Author (year)	Sample size	Cancer type	Treatment (chemotherapy regimen with mushrooms)	Control (chemotherapy-only/ mushroom-only regimen)
**Yunzhi (con't)**				
Lu et al. (2001)	112	Lung	Cyclophosphamide 600mg/m^2 IV, Doxorubicin 50 mg/m^2 IV, Cisplatin 80mg/m^2 IV on day 1 every 3 weeks or Etoposide 100 mg/m^2 IV on day 1-3, Carboplatin 300 mg/m^2 IV on day 1-3 every 3 weeks or Etopside 100mg/m^2 IV on day 1-3, Cisplatin 80 mg/m^2 IV on day 1 every 3 weeks + Xiao Ji Yin (include Yunzhi) 50 ml PO twice daily	Cyclophosphamide 600mg/m^2 IV, Doxorubicin 50 mg/m^2 IV, Cisplatin 80mg/m^2 IV on day 1 every 3 weeks or Etoposide 100 mg/m^2 IV on day 1-3, Carboplatin 300 mg/m^2 IV on day 1-3 every 3 weeks or Etopside 100mg/m^2 IV on day 1-3, Cisplatin 80 mg/m^2 IV on day 1 every 3 weeks
Maehara et al. (1984)	22	Gastric	Tegafur 400 mg IV on day 1 and 8 or 600 mg PO daily + PSK 3 g PO daily	N/A
Mitomi et al. (1992)	448	Colorectal	Mitomycin 6.0 mg/m^2 IV on day of and day after surgery, then 5-FU 200 mg/d PO + PSK 3 g/d PO	Mitomycin 6.0 mg/m^2 IV on day of and day after surgery, then 5-FU 200 mg/d PO
Miyake et al. (2018)	357	Colorectal	UFT 300 mg/m^2/d PO + PSK 3 g/d PO	UFT 300 mg/m^2/d PO, Leucovorin 75 mg/d PO
Mukai et al. (2003)	91	Colorectal	5-FU 150-300 mg PO daily or UFT 300-600 mg PO daily or 5'-DFUR 400-800 mg PO daily + PSK 3 g PO daily	5-FU 150-300 mg PO daily or UFT 300-600 mg PO daily or 5'-DFUR 400-800 mg PO daily
Mukai et al. (2003)	61	Colorectal	5-FU 150-300 mg PO daily or UFT 300-600 mg PO daily or 5'-DFUR 400-800 mg PO daily + PSK 3 g PO daily	5-FU 150-300 mg PO daily or UFT 300-600 mg PO daily or 5'-DFUR 400-800 mg PO daily
Nagao et al. (1984)	28	Leukemia	Mercaptopurine 1-2 mg/kg PO daily + Krestin 3 g PO daily	Mercaptopurine 1-2 mg/kg PO daily
Nakazato et al. (1994)	262	Gastric	Mitomycin 6 mg/m^2 on the week before Fluorouracil and PSK, Fluorouracil 150mg PO daily alternate with PSK 3g PO daily	Mitomycin 6 mg/m^2 on the week before Fluorouracil and PSK, Fluorouracil 150mg PO daily alternate with rest
Namikawa et al. (2015)	190	Gastric	S-1 40 mg/m^2 PO twice daily + PSK 1 g PO three times daily	S-1 40 mg/m^2 PO twice daily
Niimoto et al. (1988)	579	Gastric	Tegafur 600-800 mg PO daily + PSK 3g PO daily	Tegafur 600-800 mg PO daily or PSK 3g PO daily
Ogoshi & Isono (2009)	1719	Gastric	5-FU 150 mg PO daily or Tegafur 600 mg PO daily or UFT 300 mg PO daily + PSK 3 g PO daily +/- Mitomycin 0.2 mg/kg IV post-op day 1	5-FU 150 mg PO daily or Tegafur 600 mg PO daily or UFT 300 mg PO daily +/- Mitomycin 0.2 mg/kg IV post-op day 1 +/- PSK 3g PO daily
Ogoshi et al. (1994)	174	Esophageal	Futraful 600 mg PO daily + PSK 3 g PO daily (total dose over 3 months: >270g)	Futraful 600 mg PO daily or PSK 3 g PO daily (total dose over 3 months: >270g)
Ogoshi et al. (1995)	174	Esophageal	Futraful 600 mg PO daily + PSK 3 g PO daily (total dose over 3 months: >270g)	Futraful 600 mg PO daily or PSK 3 g PO daily (total dose over 3 months: >270g)
Ohwada et al. (2004)	205	Colorectal	UFT 300mg PO daily + PSK 3g PO daily	UFT 300mg PO daily
Ohwada et al. (2006)	205	Colorectal	UFT 300mg PO daily + PSK 3g PO daily	UFT 300mg PO daily
Sakai et al. (2008)	101	Colorectal	Oral fluoropyrimidine agents (UFT, carmofur and doxifluridine) 400–800 g/day + PSK 3 g PO daily	Oral fluoropyrimidine agents (UFT, carmofur and doxifluridine) 400–800 g/day
Sakamoto (1996).	262	Gastric	Mitomycin 18.2±0.2 mg IV, 5-FU PO + PSK PO	Mitomycin 18.3±0.3 mg IV, 5-FU PO

Table S3 Detailed information of the included clinical studies (con't)

Author (year)	Sample size	Cancer type	Treatment (chemotherapy regimen with mushrooms)	Control (chemotherapy-only/ mushroom-only regimen)
**Yunzhi (con't)**				
Shi & Chen. (1996)	30	Gastric	5-FU 0.5-0.75 mg IV x 5 days, Mitomycin 6-8 mg IV on day 1 + PSP 3 cap PO three times daily	5-FU 0.5-0.75 mg IV x 5 days, Mitomycin 6-8 mg IV on day 1 every month + Batilol 3 cap PO three times daily
Shiu et al. (1992)	24	Breast	Vincristine 2 mg IV on day 1, Cyclophosphamide 500 mg/m^2 IV on day 1, 4'epidoxorubicin 70 mg/m^2 IV on day 1 every 3 weeks + PSP 0.4 g 3 capsules PO three times daily (1 week prior to chemotherapy and continued for 3 more weeks after the 3rd cycle of chemotherapy)	Vincristine 2 mg IV on day 1, Cyclophosphamide 500 mg/m^2 IV on day 1, 4'epidoxorubicin 70 mg/m^2 IV on day 1 every 3 weeks
Sugimachi et al. (1997)	196	Gastric	UFT 12 mg/kg PO daily + PSK 3g PO daily	UFT 8 mg/kg PO daily, PSK 3g PO daily
Sugiyama. (1998)	11	Gastric, Colorectal	5-FU 250 mg/body/d IV, Cisplatin 5mg/body/d IV + PSK 3g/body/d PO	5-FU 250 mg/body/d IV, Cisplatin 5mg/body/d IV
Suto et al. (1994)	58	Liver	5-FU 100-150 mg PO daily + PSK 3g PO daily	5-FU 100-150 mg PO daily
Takahashi et al. (2005)	87	Colorectal	5-FU 1000 mg/m^2/24h x 2 dose IV weekly, then PSK 300 mg/day PO, alternate with 5-FU 200 mg/day	5-FU 1000 mg/m^2/24h x 2 dose IV weekly, then 5-FU 200 mg/day, alternate with no treatment
Torigoe. (1980)	20	Non-cancer	Cyclophosphamide 1 mg/kg/day PO + PSK 3 g/d PO	Cyclophosphamide 1 mg/kg/d PO ± Azathioprine 1 mg/kg/d PO
Wang et al. (2009)	64	Liver	Doxorubicin 60 mg/m^2 IV once every 21 days (not more than 550 mg/m2) + Yun Zhi Jun capsules 3 cap PO three times daily	Doxorubicin 60 mg/m^2 IV once every 21 days (not more than 550 mg/m2)
Xu et al. (2008)	53	Colorectal	Oxaliplatin 130 mg/m^2 IV, Capecitabine 2 g/m^2 IV on day 1-14 every 21 days + PSK capsule 6g/d PO	Oxaliplatin 130 mg/m^2 IV, Capecitabine 2 g/m^2 IV on day 1-14 every 21 days
Xu et al. (2003)	126	Gastric	Mitomycin 10 mg IV on day 1, FT-207 600 mg PO daily after 2 weeks + PSK 6 capsules PO daily after 2 weeks	Mitomycin 10 mg IV on day 1, FT-207 600 mg PO daily after 2 weeks
Yamagiwa et al. (1981)	169	Head and neck	5-FU 300 mg PO daily or FT-207 150-400 mg daily PO/rectal daily + PSK 3 g PO daily	5-FU 300 mg PO daily or FT-207 150-400 mg daily PO/rectal daily
Yamashita et al. (2007)	202	Colorectal	5-FU 200 mg PO daily + PSK 3 g PO daily	5-FU 200 mg PO daily
Yan & Ji. (1998)	48	Breast	Cyclophosphamide 400 mg/m^2 IV, Methotrexate 30 mg/m^2 IV, 5-FU 500 mg/m2 IV on day 1 and 8 of 4-week cycle + PSK capsules 6 cap/d PO	Cyclophosphamide 400 mg/m^2 IV, Methotrexate 30 mg/m^2 IV, 5-FU 500 mg/m^2 IV on day 1 and 8 of 4-week cycle
Yan et al. (2000)	40	Ovarian, cervical	Cisplatin 75mg/m^2 IP on day 1, Cyclophosphamide 6mg/kg/d IV on day 1-2, Doxorubicin 50 mg/m^2 IV on day 1 every 4 weeks or Mitomycin 6 mg IV on day 1 and 3, 5-FU 750 mg IV on day 1-3, Cisplatin 30 mg IV on day 1-3 every 4 weeks + PSP 0.34 g 3 capsules PO three times daily	Cisplatin 75mg/m^2 IP on day 1, Cyclophosphamide 6mg/kg/d IV on day 1-2, Doxorubicin 50 mg/m^2 IV on day 1 every 4 weeks or Mitomycin 6 mg IV on day 1 and 3, 5-FU 750 mg IV on day 1-3, Cisplatin 30 mg IV on day 1-3 every 4 weeks
Yoshitani & Takashima (2009)	63	Colorectal	UFT 400 mg PO daily + PSK 3g PO daily	UFT 400 mg PO daily
Zhang. (2010)	32	Gastric	5-FU 500 mg IV x 5 days, Doxorubicin 40 mg IV on day 1 and 3, Mitomycin 8 mg IV on day 2 and 4 + PSP capsules 0.34 g 3 cap PO three times daily	5-FU 500 mg IV x 5 days, Doxorubicin 40 mg IV on day 1 and 3, Mitomycin 8 mg IV on day 2 and 4

Table S3 Detailed information of the included clinical studies (con't)

Author (year)	Sample size	Cancer type	Treatment (chemotherapy regimen with mushrooms)	Control (chemotherapy-only/ mushroom-only regimen)
**Yunzhi (con't)**				
Zhao et al. (2004)	35	Breast	Cyclophosphamide 400 mg/m^2 IV, Methotrexate 30 mg/m^2, 5-FU 500 mg/m^2 IV on day 1 and 8 every 4 weeks + PSP capsule 2 cap PO three times daily or Selenium Yunzhi Granules 5 g PO three times daily	Cyclophosphamide 400 mg/m^2 IV, Methotrexate 30 mg/m^2, 5-FU 500 mg/m^2 IV on day 1 and 8 every 4 weeks
Zhong et al. (2001)	60	Gastric	5-FU 500-750 mg IV on day 1-5, Mitomycin 6-8mg/d on day 1 every month + PSP 1 g PO three times daily	5-FU 500-750 mg IV on day 1-5, Mitomycin 6-8 mg/d on day 1 every month + Batilol 150 µg PO three times daily

Abbreviations: IV: intravenous, PO: oral

Table S4 Detailed information of the included pre-clinical studies

Author (year)	Cell lines/ models	Mushroom (dosage)	Cytotoxic drugs (dosage)
**Lingzhi (in-vitro)**			
Cao & Lin. (2003)	Murine spleen lymphocytes	Ganoderma lucidum polysaccharides (0.2-12.8 mg/L)	Mitomycin, Etoposide (0.1 mg/L)
Chen, Ren. (2007)	Murine S-180 sarcoma	Shuang Ling Gu Ben San	5-FU, Cyclophosphamide, Mitomycin
Cui et al. (2002)	Chinese hamster lung cells V79/HGPRT	Water extract of Ganoderma lucidum (1, 10, 50 ml/L)	Mitomycin C (1 mg/L)
Feng et al. (2009)	Murine colon carcinoma cells C26	Lingzhi spore oil (0.5, 1.0 mg/mL)	Vincristine (0-10 µmol/L), Paclitaxel (0-500 nmol/L)
Guo et al. (2014)	Human bladder cancer T-24 cells	Ganoderma lucidum polysaccharides (0.125, 0.25, 0.5, 1.0, 2.0 mg/ml)	Cisplatin (0.0625, 0.125, 0.25, 0.5, 1.0 mg/L)
Huang et al. (2010)	Human urothelial carcinoma (UC) cells NTUB1; cisplatin-resistant N/P(14)	Ling-zhi polysaccharide fraction 3 (0.1, 1, 3, 10 µg/mL)	Cisplatin (14 µM)
Jiang et al. (2017)	Human colon cancer cell lines HCT-116, SW480, HT29	Ganoderma lucidum polysaccharides (200 µg/ml)	5-FU (5 µM)
Kuo et al. (2013)	Human ovarian carcinoma cell lines SKOV-3, human melanoma cell line MDA-MB-435/HER2	Ganoderma Extracts (0.25 mg/mL)	Paclitaxel (10 ng/mL), Cisplatin (10 g/mL)
Lei et al. (1993)	Murine spleen lymphocytes	Ganoderma lucidum polysaccharides (50, 100, 200 µg/ml)	Mitomycin C (0.01 µg/ml), Cytarabine (0.01 µg/ml), 5-FU (0.1 µg/ml)
Li et al. (2008)	ADM-resistant leukemic cell line K562/ADM	Ganoderma lucidum polysaccharides (5-50 mg/L)	Doxorubicin (10 mg/L)
Liang et al. (2013)	Human ovarian epithelial cancer cells HO-8910	Ganoderma triterpenes (2, 20, 200 ng/mL)	Cisplatin (2.5 µg/mL)
Liu et al. (2010)	Murine spleen lymphocytes	BW (500 µg/ mL), No. 19 (200 µg/ mL) (extract from fruiting body of Ganoderma lucidum)	5-FU (0.1, 0.5 µg/ mL)
Liu et al. (2015)	Human hepatocellular carcinoma and drug-resistance cell lines HepG2, HepG2/ADM and breast cancer and drug-resistance cell line MCF-7, MCF-7/ADR	Ganoderenic acid B (5, 10 µM)	Cisplatin, Doxorubicin, Vincristine and Paclitaxel, (IC50)
Opattova et al. (2019)	Human adherent colorectal cancer cell lines HCT116, HT29	Ganoderma lucidum (0.5 mg/ml)	5-fluorouracil (5 µM)
Qu et al. (2011)	Human ovarian cancer cells SKOV-3	Ganoderma lucidum polysaccharides (IC50)	Cisplatin (IC50)
Wang & Weng. (2006)	Human liver cells HL-7702	Ganoderma lucidum extract (0.625-5 mg/mL)	Cisplatin (2.5 µg/mL)
Xia. (2014)	HER2 Overexpressing human breast cancer cells SKBR3	Ganoderma triterpenes (12.5-100 µg/ml)	Paclitaxel (1-4 ng/ml)
Xu et al. (2009)	Human liver cancer cell lines HepG2	Ganoderma lucidum polysaccharides (1, 10, 100 µg/ml)	5-FU (50 µg/ml)
Xu et al. (2017)	Murine myocardial cells H9C2, Human breast carcinoma cells KB, oral epidermal carcinoma cells K562 and leukemia cells MCF-7	Ganoderma lucidum polysaccharides (0, 3.125, 6.25, 12.5, 25 mg/L)	Doxorubicin (1, 2, 4, 6 µmol/L)
Yao et al. (2012)	Human liver cancer cell lines HepG2	Ganoderic acid A (0-100 µM)	Cisplatin (0-10 µM)

Table S4 Detailed information of the included pre-clinical studies (con't)

Author (year)	Cell lines/ models	Mushroom (dosage)	Cytotoxic drugs (dosage)
**Lingzhi (in-vitro) (con't)**			
Yi et al. (2012)	Human colon carcinoma HCT-116 cells	Ganoderma lucidum polysaccharides (2.5, 5, 10 mg/mL)	5-FU (6.25, 12.5, 25, 50 µg/mL)
Yi et al. (2012)	Human colon carcinoma LoVo cells	Ganoderma lucidum polysaccharides (0.313, 0.625, 1.25, 2.5 mg/L)	5-FU (6.25, 12.5, 25, 50 µg/mL)
Yue et al. (2008)	Human cervical carcinoma HeLa cells CCL-2 and human non-small lung carcinoma A549 cell CCL-185	Ganoderma triterpenes (25-168.75 µg/ml)	Docetaxel, Cisplatin (5-15 nmol/L)
Yue et al. (2008)	The HeLa human cervical carcinoma cell line CCL-2	Ganoderma triterpenes (15, 50 µg/mL)	Doxorubicin (0-10 µmol/L)
Zhang & Zhang. (2016)	Human lung carcinoma A549 cells	Ganoderma lucidum polysaccharides (0.2, 0.8, 3.2 µg/mL)	Cisplatin (0.3 µg/mL)
Zhang et al. (2016)	Human breast cancer cells SKBR-3	Ganoderma triterpenes (12.5-25 µg/ml)	Paclitaxel (1-2 ng/ml)
Zhao et al. (2011)	Human epithelial ovarian cancer cell line A2780-s, A2780-cp, OV2008 and C13*	Ganoderma lucidum (0.5 mg/mL)	Cisplatin (0, 1, 2.5, 5, 10, 20 µM)
**Yunzhi (in-vitro)**			
Chan & Yeung (2006)	Human liver cancer cell lines HepG2	PSP (0.1-10 µM)	Cyclophosphamide (10 µM)
Kinoshita et al. (2010)	Human gastric cancer cell lines MKN28, MKN45, NUGC3, KATO III, AZ521, TMK-1	PSK (0-500 µg/mL)	Docetaxel (1 ng/ml)
Kobayashi et al. 1994)	Rat normal kidney fibroblasts NRK-49F and rat hepatoma H4-II-E, human ovarian cancer cells	PSK (50 µg/0.5 ml)	Cisplatin (0.1-5 µg/0.5 ml)
Ma & Xue. (1999)	Human lymphocytes	Compound Coriolus Capsules (10-50 mg/L)	Mitomycin (0.12 mg/L)
Mekata et al. (2013)	Human gastric cancer cell lines GCIY and MKN45, human colon cancer cell lines HCT116	PSK (0, 100, 300 µg/ml)	5-FU (0.3 µg/ml), 5'-deoxy-5-fluorouridine (3 µg/ml)
Rosendahl et al. (2012)	Human pancreatic cancer cell lines BxPC-3, PANC-1, MIAPaCa-2, AsPC-1	PSK (50, 500 µg/ml)	Gemcitabine (0-100 nM)
Umehara et al. (2009)	Human esophageal cancer cell lines KYSE170, TE13	PSK (0-1000 µg/mL)	5-FU (0, 0.1, 0.5, 1 µg/mL), Docetaxel (0, 0.001, 0.005, 0.01 µg/mL), Cisplatin (0, 0.5, 1.0, 5.0 µg/mL)
Wan et al. (2008)	Estrogen receptor-negative human breast cancer cells ZR-75-30	PSP (50 µg/ml)	Doxorubicin, Etoposide and Cytarabine (5 µM)
Yamasaki et al. (2009)	Human gastric adenocarcinoma cell line MK-1	PSK (0-500 µg/ml)	Docetaxel (0-100 nM)
Zhang et al. (2003)	Human pancreatic cancer cell line NOR-P1	PSK (0-500 µg/mL)	Docetaxel (1 nm)

Table S4 Detailed information of the included pre-clinical studies (con't)

Author (year)	Cell lines/ models	Mushroom (dosage)	Cytotoxic drugs (dosage)
**Lingzhi (animal)**			
Chen et al. (2005)	Hepatoma-bearing mice	Lingzhi Spore Powder (4 g/kg/d IG)	Cyclophosphamide (20 mg/kg/d IG)
Chen et al. (2008)	Sarcoma-180 bearing mice	Compound Lingzhi Spore Oil (3.7, 7.4, 14.8 g/kg IG daily)	5-FU (100mg/kg IG daily)
Chen et al. (2009)	Normal mice	Ganoderma lucidum polysaccharide (50, 100, 200 mg/kg IG daily from day 1-11)	Methotrexate (100 mg/kg IP on day 7-8)
Chen et al. (2011)	Normal mice	Ganoderma lucidum polysaccharides (50, 100, 200 mg/kg IG daily for 10 days)	Methotrexate (50 mg/kg IP on day 7 and 8)
Cui & Liu. (2002)	Normal mice	Aqueous extract of Ganoderma lucidum (3.3, 10, 30 ml/kg IG x 30 days)	Cyclophosphamide (40 mg/kg PO on last two days), Mitomycin (2mg/kg PO on day 17)
Dai et al. (2010)	Sarcoma-180 bearing mice	Ganoderma lucidum, selenium-containing Ganoderma lucidum (0.2 g/kg/d IG)	Cyclophosphamide (0.075 g/kg/d IP)
Furusawa et al. (1992)	Lewis lung carcinoma-bearing mice	Water extract from fruit bodies of Ganoderma lucidum (10 mg IP daily or on alternate days)	Doxorubicin (2 µg IP), Cisplatin (5 mg IP on day 1), Fluorouracil (0.3 mg IP on day 1), Thioguanine (0.1 mg IP on day 1), Methotrexate (0.2 mg IP on day 1), Imexon (2 mg IP day 1-6)
Gui & Wang. (1996)	Normal mice	Ganoderma lucidum injection (4 g/kg IP daily)	Cisplatin (2 mg/kg IV tail vein daily)
Gui et al. (1993)	W256 tumor-bearing mice	Ganoderma lucidum injection (1, 2, 4 g/kg IP daily)	Cisplatin (2 mg/kg IV tail vein daily)
Gui et al. (1998)	W256 tumor-bearing mice	Ganoderma lucidum injection (1, 2, 4 g/kg IP daily)	Cisplatin (2 mg/kg IV tail vein daily)
Gui et al. (1999)	W256 tumor-bearing mice	Ganoderma lucidum injection (1, 2, 4 g/kg IP daily)	Cisplatin (2 mg/kg IV tail vein daily)
Guo et al. (2014)	Human bladder cancer cell line T24-bearing mice	Ganoderma lucidum polysaccharide (200mg/kg IG daily)	Cisplatin (25mg/kg IP daily)
Guo et al. (2015)	Lewis lung carcinoma 3LL-bearing mice	Herbal Formula (include Lingzhi 0.3 g/ml) (0.4 ml/d IG for 14 days)	Cisplatin (0.1 mg/d IP on day 1, 3, 5)
Hu et al. (2003)	Sarcoma-180 bearing, murine hepatic carcinoma H22-bearing mice, dog	Compound Ganoderma lucidum Formula (5-30 ml/kg IG daily)	Cyclophosphamide (10, 84 mg/kg IP daily), Cisplatin (1, 3 mg/kg IV daily), Tegafur (80, 100 mg/kg IG daily)
Jiang et al. (2016)	Normal mice	Ganoderma lucidum polysaccharide (20 mg/kg IG daily for 15 days)	Cyclophosphamide (80 mg/kg IP daily on day 13-15)
Jiu et al. (2008)	Sarcoma-180 bearing mice	Zhenzhiling Granules (include Lingzhi) (0.071, 0.142, 0.284 g/10g IG daily)	Cyclophosphamide (0.03 g/kg IP daily)
Kashimoto et al. (2010)	Normal mice	Basal diet containing a water-soluble extract from the culture medium of Ganoderma lucidum (Reishi) mycelia (1.25, 2.5 or 5.0%) PO	5-FU (250 mg/kg IV/IP, 500 mg/kg IV), UFT (1g/kg PO), Cyclophosphamide (250 mg/kg PO, 200 mg/kg IP, 150 mg/kg SC), Cisplatin (12.5,25mg/kg IP)
Li et al. (2001)	Sarcoma-180 bearing mice	Ganoderma lucidum polysaccharide (20 mg/kg SC daily)	Cyclophosphamide (25, 50 mg/kg IG daily)
Li et al. (2011)	Sarcoma-180 bearing mice	Polysaccharide from Ganoderma (25 mg/kg PO)	Cyclophosphamide (20 mg/kg IP daily for 10 days)
Li et al. (2011)	Sarcoma-180 bearing mice	Polysaccharide from Ganoderma (25-100 mg/kg IP daily)	Cyclophosphamide (20 mg/kg IP daily for 10 days)
Li. (2012)	Human ovarian carcinoma cell lines SKOV-3 bearing mice	Cracked Ganoderma lucidum spore powder (500 mg/kg IG)	Paclitaxel (30 mg/kg/week IP)
Liu et al. (2006)	Normal mice, hepatic carcinoma H22-bearing mice	Lingzhi Spore Oil (0.625, 1.25, 2.5 g/kg IG daily for 7 days)/ Polystictus Glycopeptide Capsules (1 g/kg IG daily for 7 days)	Cyclophosphamide (100 mg/kg IP for 2 days)

Table S4 Detailed information of the included pre-clinical studies (con’t)

Author (year)	Cell lines/ models	Mushroom (dosage)	Cytotoxic drugs (dosage)
**Lingzhi (animal) (con’t)**			
Liu et al. (2007)	Normal mice	Lingqihong Capsules (including Lingzhi) (0.165, 0.825, 1.65 g/kg IG for 11 days)	Cyclophosphamide (100 mg/kg IP on day 8-10)
Liu et al. (2008)	Lewis lung carcinoma-bearing mice	Cornu Cervi Ganoderma Lucidum capsule (144 mg/kg IG daily)	Cisplatin (3.5 mg/kg IP every three days)
Liu et al. (2011)	Hepatic carcinoma H22-bearing mice	Two Polysaccharide Fractions from Ganoderma lucidum - BW (50mg/kg IP daily), GLB (50, 150 mg/kg daily)	5-FU (20mg/kg IP daily for 6 days)
Liu et al. (2015)	Human bladder cancer cell line T24-bearing mice	Ganoderma lucidum polysaccharide (200 mg /kg IG daily for 18 days)	Cisplatin (25 mg /kg IP daily for 5 days)
Liu et al. (2015)	Human bladder cancer cell line T24-bearing mice	Ganoderma lucidum polysaccharide (200mg/kg IG daily)	Cisplatin (25mg/kg IP daily)
Liu et al. (2017)	Murine hepatic carcinoma H22-bearing mice	Lingzhi Spore Oil (400, 1200 mg/kg IG daily)	Cyclophosphamide (30 mg/kg IP alternate day)
Ma et al. (2011)	Sarcoma-180 bearing mice	Selenium-containing Ganoderma lucidum mycelium (0.2 g/kg IG daily for 10 days)	Cyclophosphamide (0.075 g/kg IP daily for 10 days)
Nonaka et al. (2005)	Normal mice	2.50% Ganoderma Lucidum AF diet	Cyclophosphamide (150 mg/kg IP starting one week after AF diet)
Nonaka et al. (2008)	Normal and mammary carcinoma MM46-bearing mice	2.5% Ganoderma lucidum AF-containing AIN-93M diet	Cyclophosphamide (50, 150 mg/kg IP once)
Opattova et al. (2018)	Mouse adherent colon cancer cell line CT26.WT-bearing mice	Ganoderma lucidum (11 mg IP three times/week)	5-FU (20 mg/kg IP three times/week)
Ouyang et al. (2016)	Human lung carcinoma A549 cell-bearing mice	Ganoderma lucidum polysaccharides (50, 100, 200 mg/kg PO daily)	Cisplatin (10 mg/kg IP every two days)
Pang et al. (2007)	Murine hepatoma solidity cell HepS-bearing mice	Ganoderma lucidum polysaccharide (33.3, 100, 300 mg/kg PO daily for 9 days)	Cyclophosphamide (100 mg/kg on day 6-7)
Pillai et al. (2011)	Normal mice	Terpenes isolated from the fruiting bodies of Ganoderma lucidum (50, 100 mg/kg PO once)	Cisplatin (16 mg/kg IP once)
Rajasekaran & Kalaimagal. (2012).	Wistar rats	Ganoderma lucidum (250 mg/kg PO daily)	Doxorubicin (1.5 mg/kg IP daily)
Sha & Chan. (2018)	Human bladder cancer cell line T24-bearing mice	Ganoderma lucidum polysaccharide (IG daily)	Cisplatin (5, 25 mg/kg IG daily for 5 days)
Su et al. (2018)	Murine 4T1-breast carcinoma-bearing mice	Polysaccharide from spore of Ganoderma lucidum (200-400 mg/kg/d PO)	Paclitaxel (12.5 mg/kg IP twice weekly)
Sukegawa et al. (2017)	Rous sarcoma virus-induced S1018(B10) syngeneic tumor-bearing mice	Ganoderma Lucidum Mycelia Extract (1.5 g/kg PO daily)	Cyclophosphamide (40 mg/kg IP once)
Sun & Wei. (2001)	Normal mice	Ganoderma lucidum spore powder suspension (20, 80, 320 mg/kg IG daily)	Cyclophosphamide (40mg/kg SC daily)
Sun et al. (2007)	Sarcoma-180 bearing mice	Ganoderma lucidum extract (20 g/kg/d IG)	Cyclophosphamide (20 mg/kg/d IG)
Tang & Wu. (2013)	Sarcoma-180 bearing mice	Ganoderma lucidum mixture (50 mg/kg IG daily)	Cyclophosphamide (30 mg/kg IP alternate day)

Table S4 Detailed information of the included pre-clinical studies (con't)

Author (year)	Cell lines/ models	Mushroom (dosage)	Cytotoxic drugs (dosage)
**Lingzhi (animal) (con't)**			
Tang. (2000)	Normal mice	Aqueous extract of Ganoderma lucidum (2, 10, 20 g/kg/d IG for 8 days)	Cyclophosphamide (30 mg/kg/ SC on day 2, 3, 5)
Wang & Liang. (2016)	Human bladder cancer cell line T24-bearing mice	Ganoderma lucidum polysaccharide (200mg/kg IG daily)	Cisplatin (25mg/kg IP daily)
Wang et al. (2003)	Normal mice	Ganoderma lucidum aqueous extract (5, 10, 20 g/kg IG daily for 10 days)	Cyclophosphamide (80 mg/kg IP daily x 3)
Wang et al. (2003)	Wistar rats	Ganoderma lucidum polysaccharide (4.4, 8.8, 17.6 g/kg IP daily)	Cisplatin (2 mg/kg IV tail vein daily)
Wang et al. (2004)	W256 tumor-bearing mice	Ganoderma lucidum polysaccharide (8.8 g/kg IP daily)	Cisplatin (2 mg/kg IV tail vein daily)
Wang et al. (2004)	Wistar rats	Ganoderma lucidum polysaccharide (4.4, 8.8, 17.6 g/kg IP daily)	Cisplatin (2 mg/kg IV tail vein daily)
Wang et al. (2005)	Wistar rats	Ganoderma lucidum extract (1, 3, 10, 30 mg/kg IP x 2)	Cisplatin (3 mg/kg IP x 2)
Wang et al. (2005)	W256 tumor-bearing mice	Ganoderma lucidum polysaccharide (17.6, 35.2, 52.8 g/kg IP daily)	Cisplatin (2 mg/kg IV tail vein daily)
Watanabe et al. (2013)	Normal mice	Water soluble extract from the culture medium of Ganoderma lucidum mycelia (2.5 % (w/w) MAK PO)	5-FU (25, 80 mg/kg IP three times at 5-day intervals)
Wen et al. (1997)	Normal mice	Ganoderma lucidum mixture (200 mg/kg IG daily for 11 days)	Cyclophosphamide (100 mg/kg IP daily from day 4 for 7 days)
Wu et al. (2014)	Ehrlich ascites carcinoma tumor-bearing mice	Huo Li Su (include selenium-containing Lingzhi spore) (0.018g IG)	Cyclophosphamide (25 mg/kg IP)
Wu et al. (2017)	Ehrlich ascites carcinoma tumor-bearing mice	Huo Li Su (include selenium-containing Lingzhi spore 0.09 g/ml) (0.2 ml IG daily)	5-FU (25 mg/kg IV on day 7-9), Carboplatin (36.65 mg/kg IV on day 7-9), Epirubicin (40 mg/kg IV on day 7-9)
Xia. (2014)	Human breast carcinoma cell line SKBR-3 bearing mice	Ganoderma triterpenes (250 mg/kg PO daily for 14 days)	Paclitaxel (5 mg/kg every 3 days IV x 5)
Xie et al. (2013)	Human breast cancer cell line MCF-7 -bearing mice	Cracked Lingzhi Spore Powder (500 mg/kg IG daily for 4 weeks)	Cyclophosphamide (30 mg/kg IP weekly for 4 weeks)
Xing & Nie. (2000)	Normal mice	Oral liquid of Ganoderma lucidum polysaccharide (150, 300 mg/kg IG daily for 10 days)	Cyclophosphamide (50 mg/kg SC daily for 10 days)
Xu et al. (2017)	Normal mice	Ganoderma lucidum polysaccharide (12.5, 25, 50 µg/g IG daily)	Doxorubicin (4.5 mg/kg IP alternate day)
Yan & Bai. (2008)	Normal mice	Ganoderma lucidum polysaccharide (150 mg/kg IP daily)	Cisplatin (3mg/kg IP daily)
Yan et al. (2009)	Normal mice	Ganoderma lucidum polysaccharide (150mg/kg IP daily)	Cisplatin (3mg/kg IP daily)
Yang et al. (2015)	Human ovarian carcinoma cell lines SKOV-3 bearing mice	Cracked Ganoderma lucidum spore extract (500 mg/kg IG daily)	Paclitaxel (30 mg/kg IP once weekly)
Yang. (2008)	Normal mice	Ganoderma lucidum polysaccharide (150 mg/kg IP daily)	Cisplatin (3 mg/kg IV tail vein daily)
Yang. (2012)	Human ovarian carcinoma cell lines SKOV-3 bearing mice	Cracked Ganoderma lucidum spore powder (500 mg/kg IG)	Paclitaxel (30 mg/kg/week IP)

Table S4 Detailed information of the included pre-clinical studies (con't)

Author (year)	Cell lines/ models	Mushroom (dosage)	Cytotoxic drugs (dosage)
**Lingzhi (animal) (con't)**			
Yu et al. (2014)	Normal mice	Polysaccharide from Ganoderma (25, 50, 100 mg/kg IP from day 4-10)	Cyclophosphamide (80 mg/kg IP on day 1-3)
Zhang et al. (1997)	Normal mice	Ganoderma lucidum (62.5, 125, 250, 500 mg/kg IG daily)	Doxorubicin (3 mg/kg IP alternate day)
Zhang et al. (2006)	Pregnant mice	Ganoderma lucidum spore solution (8 g/kg/d PO twice daily)	All-trans retinoic acid (50 mg/kg PO once)
Zhang et al. (2006)	Pregnant mice	Ganoderma lucidum spore solution (8 g/kg/d PO twice daily)	All-trans retinoic acid (50 mg/kg PO once)
Zhang et al. (2012)	Sarcoma-180 bearing mice	Compounds of Ganoderma lucidum ± Astragalus extracts (250, 500, 1000 mg/kg IG daily)	Cyclophosphamide (30 mg/kg IP daily)
Zhang et al. (2014)	Murine hepatoma solidity cell HepS-bearing mice	Lingzhi Spore Oil (0.67, 1.33 g/kg IG daily)	Cyclophosphamide (15, 30 mg/kg IG daily)
Zhang et al. (2016)	Human breast carcinoma cell line SKBR-3 bearing mice	Ganoderma triterpenes (250 mg/kg PO daily for 14 days)	Paclitaxel (5 mg/kg every 3 days IV x 5)
Zhao et al. (2012)	Lewis lung carcinoma-bearing mice	Ganoderma lucidum polysaccharide (40 mg/kg IG every two days)	Cisplatin (2mg/kg IP every two days)
Zhou et al. (2009)	Hepatic carcinoma H22-bearing mice	Ganoderma lucidum polysaccharide (1.02 g/kg IG daily for 12 days)	Cyclophosphamide (100 mg/kg IP on day 1 and 6)
Zhou et al. (2013)	Normal mice	Recombinant Ganoderma lucidum immunoregulatory protein (160 µg/kg/d IP twice daily on selected days)	Cyclophosphamide (50, 165 mg/kg/d IP on selected days)
Zhou et al. (2015)	Chicken immunized with Newcastle disease vaccine	Oral liquid of Astragalus and Lucidum polysaccharides (include Ganoderma lucidum polysacchardies 0.5, 2.5, 5 mg/kg PO for 5 days)	Cyclophosphamide (80 mg/kg SC for 3 days)
Zhu et al. (2007)	Normal Mice	Ganoderma lucidum polysaccharides (2.5, 25, 250 mg/kg IP for 7 days)	Cyclophosphamide (300 mg/kg IP once)
Zhu et al. (2019)	Mouse cervical carcinoma U14-bearing mice	Ganoderma lucidum polysaccharides (30 mg/kg IV daily)	Cisplatin (5 mg/kg IV daily)
**Yunzhi (animal)**			
Chan, & Yeung. (2006)	Sprague Dawley rats	PSK (2, 4 µmol/kg/d IP)	Cyclophosphamide (358 mmol/kg IV x 1 dose)
Deng et al. (1985)	Mice Immunized With IgG Anti-Sheep Red Blood Cells (SRBC)	PSK (25-50 mg/kg IP)	Cyclophosphamide (15-100 mg/kg IH/IP), 6-mercaptopurine (100 mg/kg PO), Methotrexate (1.5 mg/kg IP), 5-FU (20, 25 mg/kg IP), Mitomycin (2 mg/kg IP)
Fujii et al. (1989)	Sarcoma-180 or X5563 tumor-bearing mice	Krestin (50 mg/kg IP 3 times per week or 1g/kg PO daily)	Mitomycin (1 mg/kg IP 4 times in first week, 0.25 mg/kg IP in 3rd week or 0.25 mg/kg IV 3 times on 1st and 3rd week)
Fujita et al. (1988)	Sarcoma 180-bearing mice	PSK (400 mg/kg/d PO or 100 mg/kg/d IV for 4 days)	Tegafur (400 mg/kg 24 hours after PSK)
Garrido et al. (2011)	Mouse fibrosarcoma GR9-A7-bearing mice	PSK (2.5 mg/week IP)	Docetaxel (125 µ/week IP)
Guo et al. (2012)	Human lung carcinoma A549-bearing mice	Coriolus versicolor polysaccharides (60 mg/kg/d IP)	Cisplatin (40 mg/kg IP three times weekly)
Hasegawa et al. (1989)	Normal mice	Krestin (75, 150, 300 mg/kg IP)	Mitomycin (2 mg/kg IV)

Table S4 Detailed information of the included pre-clinical studies (con't)

Author (year)	Cell lines/ models	Mushroom (dosage)	Cytotoxic drugs (dosage)
**Yunzhi (animal) (con't)**			
Hosokawa et al. (1985)	3-methylcholanthrene induced tumor-bearing C57BL/6 Mice	PSK (300 mg/kg/d for 5 days or twice weekly x 7 weeks IP)	Busulfan (50mg/kg PO on the day of tumor removal followed by two further dose weekly x 3 weeks)
Iwaguchi et al. (1989)	Plasmacytoma X5563-bearing mice	PSK (10 mg/kg IP every other day)	Mitomycin C (1 mg/kg IP every other day)
Jin et al. (2008)	Ehrlich's ascites carcinoma-bearing mice	PSP (250 mg/kg/d IG)	Doxorubicin (5.33 mg/kg/d IP x 3 days)
Katoh & Ooshiro. (2007)	Mouse Meth A sarcoma, mouse Lewis lung carcinoma and mouse colon 26 carcinoma-bearing mice	PSK (5, 10, 50, 100 mg/kg IP 3 times per week on day 1-28)	UFT (60 mg/kg PO from day 1-9), Leucovorin (5.56-22.4 mg/kg PO from day 1-9)
Katoh. (1998)	Lung cancer LC-10 tumor-bearing mice	PSK (1 g/kg PO)	Carboplatin (10 mg/kg IV on day 1), Etoposide (30 mg/kg IV for 3 days)
Kinoshita et al. (2010)	Severe-combined immunodeficient mice (CB17 SCID)	PSK (50 mg/kg IP 3 times every week)	Docetaxel (0-20.0 mg/kg IV on 14th and 21st day)
Li et al. (2008)	Ehrlich ascites carcinoma-bearing mice	PSP (250 mg/kg/d IG)	Doxorubicin (4 mg/kg/d IP x 3 days)
Li et al. (2009)	Normal mice	Sanghuang Yunzhi Capsule (4, 8 g/kg IG daily)	Cyclophosphamide (80 mg/kg IP on day 4-6), Cisplatin (7mg/kg on day 4 IP)
Li. (2008)	Ehrlich ascites carcinoma-bearing mice	PSP (250 mg/kg/d IG)	Doxorubicin (4 mg/kg IP x 3 days)
Liu et al. (2006)	Normal mice and murine hepatic carcinoma H22-bearing mice	Lingzhi Spore Oil (0.625, 1.25, 2.5 g/kg IG), Polystictus Glycopeptide Capsules (1 g/kg IG)	Cyclophosphamide (100 mg/kg IP)
Mizushima et al. (1982)	Fibrosarcoma-bearing rat and mice	PSK (50 mg IP on Days 3 and 4 or 6 and 7)	Cyclophosphamide (0-100 mg/kg IP on Day 5)
Nie. (2009)	Murine hepatic carcinoma H22-bearing mice	Sanghuang Yunzhi Capsule (4, 8, 16 g/kg IG daily)	5-FU (0.03 g/kg IG daily)
Oh-hashi et al. (1978)	Leukemia P388 cell-bearing CD2F1 mice	PSK (500 or 1000 mg/kg IP on day 2,5 or 8 (with Mitomycin) and on day 5 (with other drugs))	Mitomycin (1 mg/kg IP), 5-FU (50 mg/kg IP), 6-mercaptopurine (100 mg/kg IP), Cyclophosphamide (12.5 mg/kg IP) on day 1
Qian et al. (1997)	Wistar rats	PSP (2 g/kg/d PO)	Cyclophosphamide (40 mg/kg every 2 days PO)
Shen et al. (2008)	Sarcoma-180 bearing mice	PSP (200, 400, 800 mg/kg IG daily)	Cyclophosphamide (40 mg/kg IG or 30 mg/kg IP daily)
Takenoshita et al. (1995)	Hepatocellular carcinoma KDH-8 bearing rats	PSK (1000 mg/kg PO 5 times/week)	5-FU (12.5 mg/kg PO 5 times/week)
Tsuru et al. (1991)	Liver metastatic tumor-bearing mice	PSK (1 g/kg/day PO daily)	Tegafur (117, 176 mg/kg/d PO daily), 5-FU (35 mg/kg/d PO daily)
Ueno et al. (1994)	B-16 melanoma-bearing mice	PSK (1 g/kg PO daily)	Cyclophosphamide (20 mg/kg IP on selected days)
Wenner et al. (2012)	Mouse prostate tumor (TRAMP-C2) bearing mice	PSK (300 mg/kg PO daily)	Docetaxel (5 mg/kg IP twice weekly)
Wu et al. (2015)	Normal mice	PSP (125 mg/kg IG daily)	Cyclophosphamide (150 mg/kg IP on day 17 and 21)
Yamasaki et al. (2009)	Gastric adenocarcinoma MK-1-bearing mice	PSK (50 mg/kg IP 3 times every week)	Docetaxel (2.5 or 5 mg/kg IV on days 14 and 21)
Zhang et al. (2013)	Normal mice	PSP (125 mg/kg IG daily)	Cyclophosphamide (150 mg/kg IP on day 17 and 21)
Zhang et al. (2013)	Normal mice	PSP (125, 500 mg/kg IG daily)	Cyclophosphamide (150 mg/kg IP on day 17 and 21)
Zhu et al. (2008)	Ehrlich ascites carcinoma-bearing mice	PSP (250 mg/kg/d IG), Tanshinone (50 mg/kg/d IG)	Doxorubicin (4 mg/kg/d IP)
Zhu et al. (2008)	Ehrlich ascites carcinoma-bearing mice	PSP (250 mg/kg/d IG), Tanshinone (50 mg/kg/d IG)	Doxorubicin (4 mg/kg/d IP)

Abbreviations: IG: intragstric, IP: intraperitoneal, IV: intravenous, PO: oral

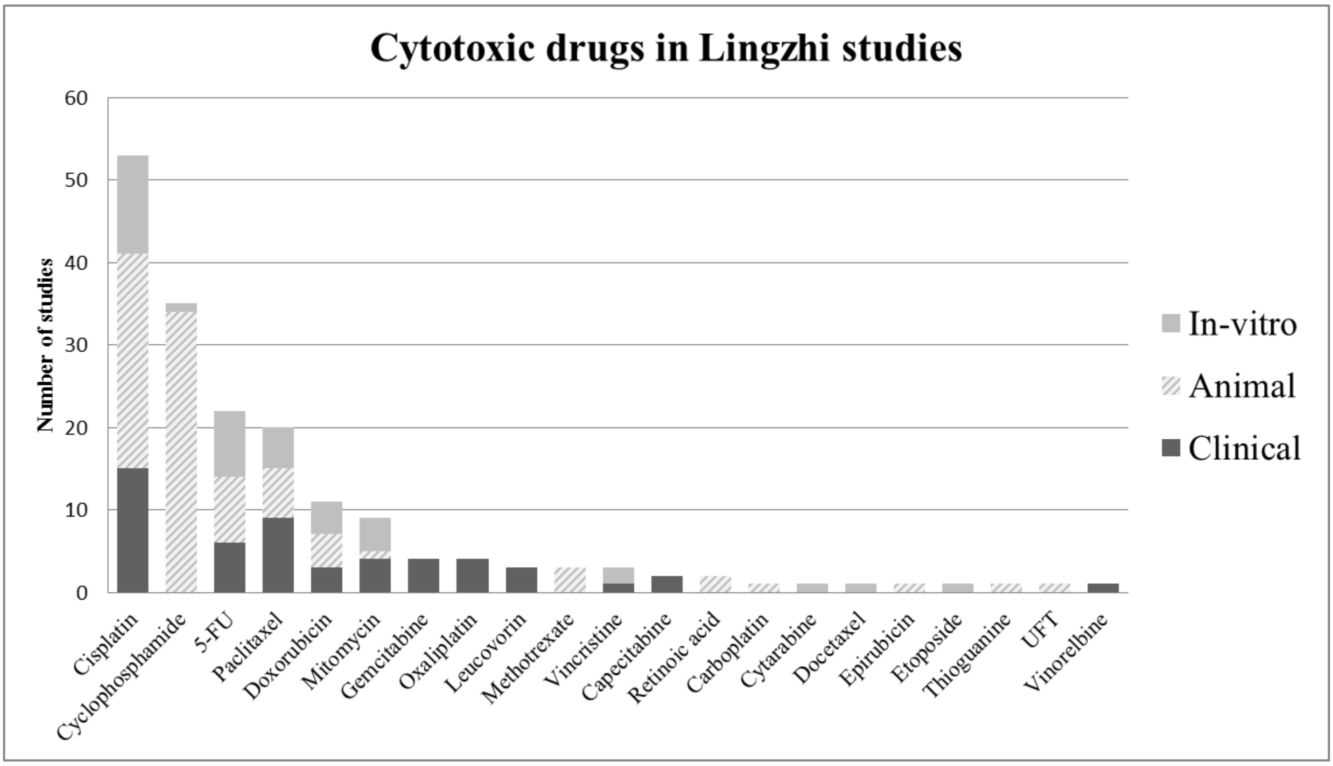

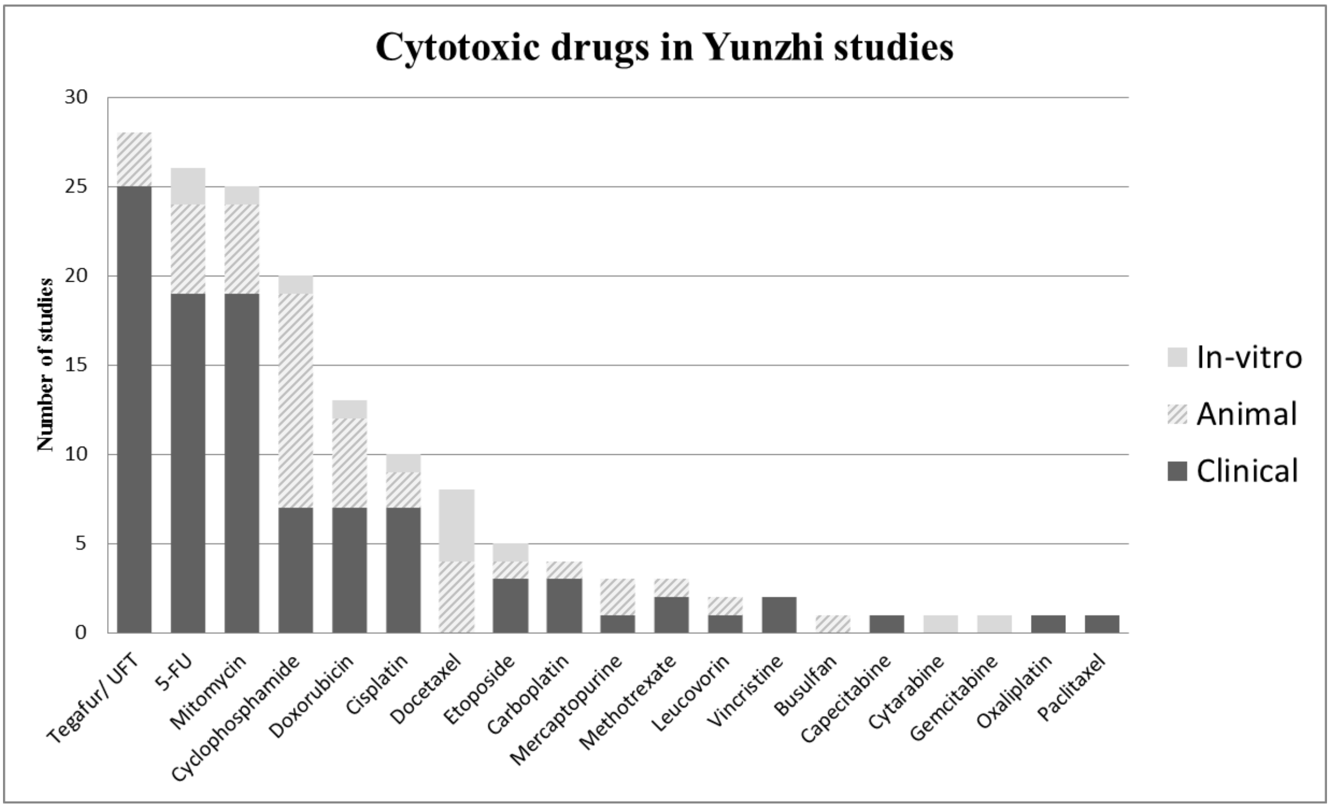

Figure S1 **Distribution of pre-clinical and clinical studies for combination use of cytotoxic drugs with Lingzhi and Yunzhi.**
